# Optoelectronic simulation and optimization of all perovskites tandem solar cells employing electrodeposited copper oxide as hole transport layer

**DOI:** 10.1038/s41598-025-93982-7

**Published:** 2025-03-22

**Authors:** Vishwas D. Patel, Dhritiman Gupta

**Affiliations:** Department of Physics, School of Advanced Sciences (SAS), Vellore Institute of Technology (VIT), Vellore, Tamil Nadu 632014 India

**Keywords:** Electrodeposited Cu_2_O film, Hole transport layer, 2T tandem solar cell, 4T tandem solar cell, Energy harvesting, Renewable energy, Electronics, photonics and device physics, Materials for devices, Materials for energy and catalysis

## Abstract

Tandem solar cells are highly promising photovoltaic device that can potentially beat the maximum power conversion efficiency achieved so far in a single junction silicon solar cell by mitigating both the thermalization and transmission losses commonly encountered in a single junction solar cell. Among several different components of a tandem solar cell, hole-transport layer (HTL) plays an important role. Present day state of the art HTL layers are limited in number and sometimes highly expensive. In this work, we explore the feasibility of using electrodeposited Cu_2_O and mixed phase (Cu_2_O + CuO) Cu–O film as HTL in all-perovskite tandem solar cells and a detail optical, compositional and morphological analysis was performed. To access its performance as HTL in tandem devices, here we adapted an optoelectronic simulation approach using SCAPS-1D software tool and transfer matrix simulation routine where the parameters were either measured experimentally or carefully optimized to replicate the performance under realistic testing conditions. Photovoltaic parameters for single-junction cells with Cu_2_O-HTL was found to be less sensitive on the electron affinity of the Cu_2_O as opposed to that of Cu–O in a Cu–O-HTL based single junction cells. The highest efficiency predicted in our simulation is 24.95% in a 2-terminal tandem device with Cu_2_O-HTL and electron affinity of 3.8 eV whereas with similar device architecture, in a 4-terminal tandem device, the highest efficiency can reach upto 35%.

## Introduction

The seminal paper of Schockley and Queasier (SQ) predicted the maximum theoretical efficiency for a solar cell to be 33% in a single junction device and with an optimum optical bandgap (${E}_{g}$) of 1.45 eV for the light absorbing medium^1^. Tandem and multijunction solar cells are envisaged as promising approaches to beat the SQ limit. Using a detailed balance limit calculation it has been predicted that efficiency can reach 42% in a double junction (or tandem) and 49% in a triple junction solar cell^2^. In addition, in a multiple junction solar cell, the efficiency can reach up to ~ 65%^3^. These predicted values are quite encouraging, and researchers have successfully fabricated multijunction solar cell devices which have surpassed the 30% efficiency benchmark, the SQ limit for a single junction cell^10^. Monolithic, two-terminal (2T), double junction or tandem solar cells are constructed by stacking two solar cells atop each other, one with a narrow-bandgap (NBG) absorber layer (as back sub-cell) and the other with a wide-bandgap (WBG) absorber layer (as front sub-cell). Light enters the device through front sub-cell and the back subcell receives the filtered light. In between these two cells, there are charge recombination layer which is a combination of hole-transport layer (HTL) and electron-transport layer (ETL). In a four-terminal (4T) tandem device, the intermediate charge recombination layer can be omitted by placing two sub-cells on top each other mechanically. This design is not a monolithic structure, however, 4T device structure does not require current matching between two sub cells and the resultant power conversion efficiency (PCE) is just the sum of PCE values of two sub cells. After the breakthrough in perovskite-based solar cell research, scientists across the world have explored several combinations of sub-cells in conjunction with perovskite sub cell, namely, perovskite/silicon, perovskite/CIGS, perovskite/perovskite and perovskite/organic^4–8^. The certified power PCE of perovskite/silicon and perovskite/CIGS (Glass/Molybdenum/CIGS ($E_{g} \approx 1.1 \;{\text{eV}}$)/CdS/ ZnO/SAM/Cs_0.05_(MA_0.23_FA_0.77_)Pb_1.1_(I_0.77_Br_0.23_)_3_ (${E}_{g}\approx 1.68\, {\text{eV}}$)/C_60_/SnO_2_/IZO/LiF) 2T tandem solar cells are 34.6% ^9,10^and 24.2%^11^ respectively whereas that for perovskite/organic tandem device, (ITO/NiO_x_/2PACz/FA_0.8_Cs_0.2_Pb(I_0.5_Br_0.5_)_3_/C_60_/BCP/Au/MoO_x_/ PM6:PM7: Y6:PC_71_BM/C_60_/BCP/Ag) is 25.1%^12,13^. On the other hand, the highest PCE for perovskite/perovskite tandem device (PPTSC) recorded so far is 30.1% in a 2T configuration^10,14^. Large area (~ 1 cm^2^) 4T-tandem based on WBG-perovskite, FA_0.7_Cs_0.25_MA_0.05_Pb(I_0.8_Br_0.2_)_3_ (${E}_{g}\approx 1.67 \,\text{eV}$) as front sub-cell and NBG-perovskite, FA_0.7_MA_0.3_Pb_0.5_Sn_0.5_I_3_ with ${E}_{g}\approx 1.25$ eV as back sub-cell has been reported by Peng et. al. with a PCE of 28.35%^15^. Among solution processable devices, PPTSCs seem to offer an edge over the perovskite/organic combination due the similarity and compatibility in process conditions. It has witnessed a rapid increase in PCE in a short span of time from 24.2% (glass/ITO/NiO/FA_0.8_Cs_0.2_Pb(I_0.6_Br_0.4_)_3_ (${E}_{g}\approx 1.77 \,\text{eV}$) /C_60_/SnO_2_/Au/PEDOTPS /FA_0.7_MA_0.3_Pb_0.5_Sn_0.5_I_3_(${E}_{g}\approx 1.22 \,\text{eV}$)/C_60_/SnO_2_/Cu)^16^ in 2019 to 26.4% ^14^ in 2021, with a modified device configuration (Glass/ITO/NiO/VNPB/ FA_0.8_Cs_0.2_Pb(I_0.62_Br_0.38_)_3_ (${E}_{g}\approx 1.76\,\text{ eV},\text{WBG}$))/C_60_/SnO_2_/Au/ PEDOT: PSS/ FA_0.7_MA_0.3_Pb_0.5_Sn_0.5_I_3_ (${E}_{g}\approx 1.22\,\text{ eV},\text{NBG}$)/C_60_/BCP/Cu). Both the cases, the device area was 1.041cm^2^. Recently, in the year 2022, this PCE was revised to 29.1% by the same group with smaller device area of 0.0494 cm^−2^. For further development and progress in PCE, there is a need for developing new perovskite materials along with low-cost, environmentally stable charge transport layers. HTL and ETL form the intermediate layer between perovskite and metal contacts. The combination of HTL and ETL form the charge recombination layer in tandem devices. The widely used HTL is PEDOT: PSS (polyethylene dioxythiophene: polystyrene sulfonate) and PTAA (poly[bis(4-phenyl)(2,4,6-trimethylphenyl)amine]). In some cases, Spiro-OMeTAD is used as HTL. However, the complex synthesis and purification process makes them so expensive that it is almost impractical to use them at the commercialization stage^17,18^. PEDOT:PSS is water based formulation and slightly acidic which affects the device stability in the long run. However, metal oxides are cheap and environment-friendly materials that can also be solution processed. Zinc oxide and titanium di oxide are two highly successful n-type materials for ETL, however, the number of p-type oxides with wide bandgap for HTL applications are limited. So far, nickel oxide has been used widely as HTL material with some success. The PCE values for NiO_x_ HTL based tandem devices has witnessed an upward trend, 16.9%^19^ in 2016 to a certified efficiency of 27.04%^20^ in 2024. However, in literature, it has been argued that the interface defect states and undesired chemical reactions at the NiO/perovskite interface may limit the PCE values. Hence it has become essential to further explore the feasibility of other p-type oxides such as oxides of copper (CuO and Cu_2_O) as HTL material.

The perovskite solar cell (ITO/Cu_2_O/CH_3_NH_3_PbI_3_/PCBM/Ag) was fabricated by Weili, et al. ^21^ using Cu_2_O as HTL. The Cu_2_O film was prepared using thermal oxidation of vacuum evaporated Cu layer, in ambient condition at 250 °C. However, XPS analysis confirmed the presence of both Cu^1+^ and Cu^2+^ with 86% and 14% atomic percentages respectively. Solution processible SILAR technique was demonstrated by Chatterjee et al.^22^ for the preparation of Cu_2_O film and used for the device fabrication (ITO/Cu_2_O/CH_3_NH_3_PbI_3−x_Cl_X_/PCBM/Al). However, there was no evidence of purity of the film in terms of X-ray diffraction (XRD) or X-ray photoelectron spectroscopy (XPS). Zuo and Ding^23^ fabricated perovskite solar cells (ITO/HTL/CH_3_NH_3_PbI_3_/PC_61_BM/Al) using Cu_2_O and CuO as HTL. They have presented a unique low-temperature conversion method to prepare a pure form of Cu_2_O and CuO films. In the process, solution of CuI film was coated on ITO and further reacted with NaOH. Thus, the prepared film was annealed at 100 °C for 10 min in glovebox to obtain Cu_2_O phase. Further annealing the prepared Cu_2_O film at 250 °C in air yields CuO phase. The phase of the prepared films was analysed using XRD, however no evidence in terms of XPS data was presented. Rao, Haixia, et al.^24^ and Sun, Weihai, et al.^25^ fabricated perovskite solar cell using non-stoichiometric copper oxide films. The mixed phase of the film was studied using XPS.

Simulation of single junction perovskite solar cell was carried by Hossain et al.^26^, using SCAPS-1D and wxAMPS to understand the best device performance of CsPbI_3_ based perovskite solar cell. Authors have used different HTL (Cu_2_O, CuSCN, CuSbS_2_, NiO, P3HT, PEDOT:PSS, Spiro-MeOTAD, CuI, CuO, V_2_O_5_, CBTS, and CFTS) and ETL layers(PCBM, TiO_2_, ZnO, C_60_, IGZO, SnO_2_, WS_2_, and CeO_2_) for the simulation process. The input parameters for ETLs and HTLs were adapted from the literature data whereas for CsPbI_3_ they have used DFT simulation. Baro et al.^27^ and Jayan et al.^28^ simulated the perovskite solar cell with multiple HTL and ELT layers using SCAPS-1D. For the simulation of the device the input data were borrowed from the literature for all the layers and the simulated data was not compared with any preexisting experimental results. However, Zhao, Qirong et al.^29^ simulated device with the architecture FTO/TiO_2_/MASnI_3_/Cu_2_O/Au using experimental data of Cu_2_O film. In their study they have prepared Cu_2_O film via solution processed technique by annealing it at 388 °C. The valence band edge was calculated using XPS analysis and used the data for the simulation of the device.

All perovskite tandem solar cell (ITO/Cu_2_O/FA_0.8_Cs_0.2_Pb(I_0.7_Br_0.3_)_3_(Wide Bandgap)/PCBM/SnO_2_/ITO/PEDOT:PSS/(FASnI_3_)_0.6_(MAPbI_3_)_0.4_:Cl(Narrow Bandgap)/PCBM/SnO_2_/Ag) was simulated by Shankar et. al.^30^. SCAPS-1D was used for the simulation of 2T tandem device where the input parameters were picked from the literature. In the simulation they have varied the thickness of the perovskite active layer to achieve the best device efficiency. Similarly, Singh et. al.^31^ have simulated 2T all perovskite tandem solar cell (FTO/ZnO/CH_3_NH_3_GeI_3_** (**Wide Bandgap**)** /p^+^/n^+^/FAMASnGeI_3_ (Narrow Bandgap)/Cu_2_O/Au using SCAPS-1D. In this case also, all input parameters were adapted from literature without any subsequent comparison with experimental data. Ahmed et al.^32^ and Hossain et al.^33^ simulated 4T tandem solar cell using SCAPS-1D. They have mainly focused on optimizing the perovskite layer by varying the thickness to achieve the best results. In both the work the all the input parameters for all the layers were adapted from the published literature.

As the race for high efficiency tandem device is intensifying, researchers are trying to assess the potential of new materials as fast as possible. A combined approach of simulation and experiment is therefore preferred as opposed to only experimental one. Optoelectronic simulation has the advantage of predicting the realistic estimate of PCE value without having to make the device and test it. Undoubtedly, the experimental PCE value gets the ultimate recognition. A close match between experimentally published results and simulated results therefore is desirable since it points towards the reliability of the simulation conditions and parameters. Despite the limitations to replicate realistic conditions in terms of materials parameters and testing conditions, many simulation-oriented research article has been published for tandem solar cells to predict the potential it holds for future^34,35^. Most of the simulation-only papers predicted PCE > 30% in PPTSC devices. By optimizing the optical constants for maximizing the light coupling into the subcells and combining it with analytical current/voltage characteristics, Soldera et al. has predicted an PCE value of 37% in 2T PPTSC^36^. Hörantner et al. used an optical (Transfer matrix method) and electrical model (to simulate double and multijunction perovskite-based solar cells^37^. The group has predicted an efficiency of 33.4% efficiency for all perovskite double junction solar cell. Similarly, Yadav et al. on tailoring of the perovskite active layer properties such as thickness, resistance, trap density the charge carrier mobilities have increased the PCE > 30%^38^. Madan, J et al. simulated the device using SCAPS-1D where they focused on varying the thickness of active layers and the use of lead-free perovskite as one of the subcells^49^. In supplementary Figure S1a and S1b we have summarized the PCE of experimental and simulated 2T and 4T tandem solar cell values based on metal oxide HTL layer, reported over last 5 years along with the calculated efficiency obtained in this work.

In this research article we adopted a combined approach of experiment and optoelectronic simulation to demonstrate the potential of CuO and Cu_2_O as two promising HTL material. Pure phase Cu_2_O was deposited through electrodeposition process, and it was converted to mixed phase (CuO + Cu_2_O) film through annealing in air. Both the films were characterized using optical, morphological and elemental analysis techniques. Bandgap and the relative positions of conduction and valence bands were extracted from the UPS measurements. To assess the performance of these films as HTL in PTSC, we have done a detail optical and electrical simulation-based analysis using transfer matrix method and SCAPS-1D simulation platform. The device structure for PPTSC was based on combination of optimized bandgap perovskite materials namely, HBG-perovskite, FA_0.8_Cs_0.2_Pb(I_0.6_Br_0.4_)_3_ with ${E}_{g}\sim 1.77 \,{\text{ eV}}$ as front-subcell and NBG-perovskite, FA_0.7_MA_0.3_Pb_0.5_Sn_0.5_I with ${E}_{g}\sim 1.22 \,{\text{ eV}}$ as back sub-cell. The complete tandem architecture mimics the device geometry of Xio et al.^16^ (glass/ITO/NiO_x_/VNPB/ FA_0.8_Cs_0.2_Pb(I_0.6_Br_0.4_)_3_/C_60_/SnO_2_/Au/PEDOTPS/FA_0.7_MA_0.3_Pb_0.5_Sn_0.5_I_3_/C_60_/SnO_2_/Cu) which was studied experimentally and was report to exhibit a certified efficiency of 24.2%^16^. However, to prove the efficacy of the synthesized copper oxide films as HTL, the NiO_x_ layer has been replaced by Cu–O and Cu_2_O, keeping all other layer components unaltered. To optimize the device parameters, first two standalone single junction devices are simulated using SCAPS-1D and a close match with experimentally reported $J-V$ characteristics were obtained by tuning parameter values. In the next step, a tandem device was constructed, and thickness optimization of front and back cell was performed using a combined optical and electrical SCAPS-1D simulation. The PCE values were found to have strong dependence on the electron affinity of Cu–O. With experimentally obtained value of electron affinity ($\approx 2.84-2.86{\text{V}}$), the PCE for Cu–O-HTL 2T PPTSC was $18.54\%$ and that for Cu_2_O HTL-based 2T device was $24.15\%$. While for 4T devices, the PCE was $32.276\%$ and $34.98\%$ for Cu–O and Cu_2_O-HTL, respectively. When electron affinity of Cu–O and Cu_2_O is increased to $3.80 \,{\text{ eV}}$, the corresponding 2T tandem device efficiency was raised up to 24.27% and 24.95% respectively. However, 4T tandem devices with Cu_2_O and Cu–O as HTL exhibit efficiency of $35.51\%$ and 39.16%, respectively.

In this work, we show how to combine SCAPS-1D with TMM to simulate a truly monolithic 2T tandem device with more than 7 layers. Employing this unique methodology, we show a comparison between pure Cu_2_O and mixed Cu–O as HTL. All the optical parameters for copper oxide layer, used in this simulation, was measured experimentally enhancing the reliability of the simulated data. UPS measurement on copper oxide was performed to gain a realistic estimate to the energy levels. Quite interestingly, pure Cu_2_O HTL not only yield better PCE values, but it is also inert to the changes in electron affinity. Cu–O only slightly outperforms the Cu_2_O HTL for higher electron affinity values.

## Experimental

### Materials

All chemicals used in the experiment were of a high standard of purity. Copper (II) sulphate (CuSO_4_) anhydrous (99%, HIMEDIA), Lactic acid (C_3_H_6_O_3_) (Hi-AR™/ACS, HIMEDIA), Sodium hydroxide (NaOH) (98%, SRL) was purchased and used as received. ITO substrates were purchased from Ossila with a sheet resistance of 20 $\Omega /{\text{sq}}$ and RMS roughness of 1.8 nm.

### Preparation of Cu_2_O and Cu–O thin films

For electrodeposition of copper oxide, indium tin oxide (ITO) coated glass substrate was used as working electrode in a conventional three-electrode cell configuration (CHI-601C Electrochemical Workstation). Before the deposition, the ITO substrates were thoroughly cleaned with soap solution followed by cleaning with DI (deionized) water using an ultrasonicator. Further, the substrates were sonicated in acetone and isopropyl alcohol (IPA) for 15 min each then dried and treated with UV–ozone (Holmarc, India) for 30 min. Cu_2_O layer was deposited on a precleaned ITO substrate (working electrode) using a constant applied potential of $- 0.3 {\text{V}}$ vs. Ag/AgCl (reference electrode) at room temperature and Platinum (Pt) wire was used as the counter electrode in the process^39^ (Supplementary Figure S2a). For the deposition of Cu_2_O film, electrolyte solution was prepared using 0.4 M ${\text{Cu}}{\text{SO}}_{4}$ and 3 M lactic acid (${\text{C}}_{3}{{\text{H}}}_{6}{{\text{O}}}_{3}$) in deionized water. It resulted in a pale-blue colour solution with a pH of 3. In the next step, 9 M NaOH solution was added dropwise to the prepared CuSO_4_ solution until it reached the pH of 11 and the colour of the solution turned to violet. Higher level of pH ($>7$) ensures the p-type conductivity in Cu_2_O while the acidic nature of the electrolyte solution (pH range from 4.5—5.5) was reported to facilitate the growth of n-type Cu_2_O thin films^40^. Each time, a freshly prepared solution with pH maintained at 11 was used for electrodeposition. Chemical reactions involved in formation of Cu_2_O on the ITO electrode are; (i) ${\text{Cu}}^{{2 + }} + {\text{2e}}^{ - } \to {\text{ Cu}}$ (Reduction reaction at the ITO substrate),

(ii) ${\text{C}}_{3} {\text{H}}_{6} {\text{O}}_{3} \to 2{\text{CO}}_{2} {\text{ + 2H}}^{ + } {\text{ + }}2{\text{e}}^{ - }$ (Oxidation reaction at the counter (Platinum) electrode), (iii) ${2\text{Cu}}^{+}+{2\text{OH}}^{-}\to {\text{Cu}}_{2}\text{O}+{\text{H}}_{2}\text{O}$ (Formation of Cu_2_O). Lactic acid ${\text{(C}}_{3}{{\text{H}}}_{6}{{\text{O}}}_{3})$ undergoes oxidation process at counter electrode and facilitates the transport of Cu^2+^ towards ITO. The coated pure-phase Cu_2_O films were thoroughly rinsed with DI water to clean up the film surface in contact with the electrolyte solution, since any residual unreacted species can affect the phase formation and degrade the coated layer. Films thus obtained were annealed in vacuum at 100 °C to remove the volatile surface contaminants. To obtain Cu–O films, the Cu_2_O films were annealed at 300 °C in the air. It led to the transition of Cu_2_O to mixed phase of copper oxide (Cu_2_O + CuO). The prepared films showed a compact uniform surface with a particle size of 92 nm and 138 nm for Cu_2_O and mixed phase film respectively (Supplementary Figure S2b and S2c). Throughout the rest of the manuscript, this mixed phase film will be referred as Cu–O.

### Device architecture and simulation methodology

**Fig. 1:**
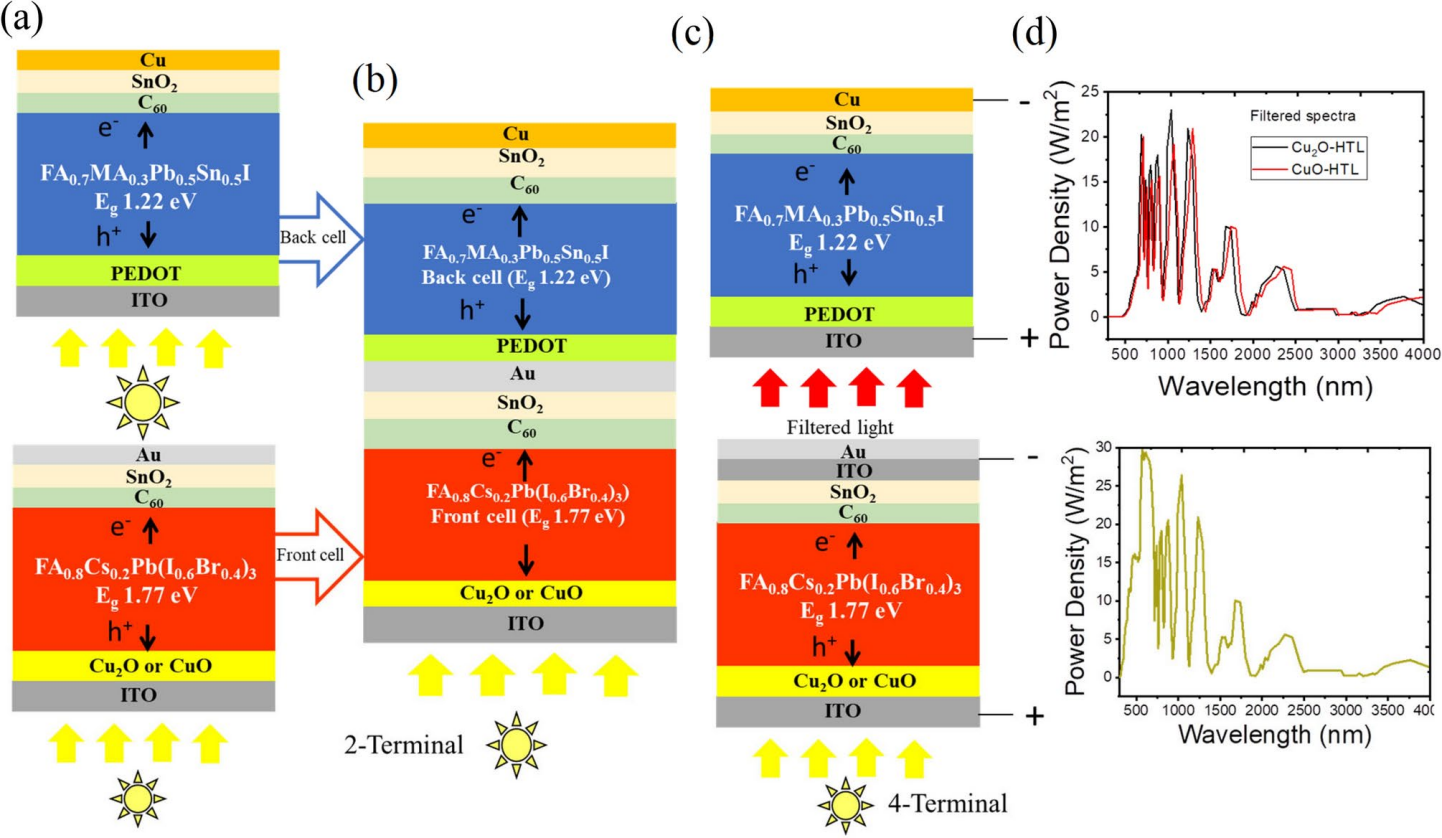
Schematic representation of (**a**) single junction WBG-perovskite, FA_0.8_Cs_0.2_Pb(I_0.6_Br_0.4_)_3_ ${(E}_{g}\approx 1.77 \,{\text{ eV}})$ device with Cu_2_O or CuO as HTL layer and single junction NBG-perovskite, FA_0.7_MA_0.3_Pb_0.5_Sn_0.5_I (${E}_{g}\approx 1.22 \,{\text{ eV}})$ device with PEDOT: PSS as HTL layer. All-perovskite/perovskite tandem solar cell (PPTSC) device in (**b**) 2-terminal device architecture and (**c**) 4-terminal device architecture. (**d**) AM1.5G spectra received by front cell and filtered spectra received by back cell.

To study the effectiveness of the electrodeposited copper oxide layers as HTL in tandem solar cell, we adapted a simulation-based approach wherein the device architecture was chosen to be same as the 2T tandem device reported by Xiao et al.^16^ The key difference here is that the NiO_X_ HTL layer has been replaced by Cu_2_O and Cu–O. The complete device architecture (glass/ITO/Cu_2_O or Cu–O /FA_0.8_Cs_0.2_Pb(I_0.6_Br_0.4_)_3_ ${(E}_{g}\approx 1.77 \,\text{eV},\text{WBG})$/C_60_/SnO_2_/Au/PEDOT: PSS/FA_0.7_MA_0.3_Pb_0.5_Sn_0.5_I ${(E}_{g}\approx 1.22 \,\text{eV},\text{NBG})$ /C_60_/SnO_2_/Cu)) has been shown in Fig. 1a–c. We have considered both a 2T tandem as well as a 4T tandem device. The simulation was carried out by an open-source tool SCAPS 1D (Solar Cell Capacitance Simulator) version 3.3.10 developed at the University of Ghent (Department of electronics and information Systems (ELIS)), Belgium^41^. It is a one-dimensional simulation tool that involves Poisson’s (Eq. 1) and continuity equations^42^ (Eqs. 2, 3) which need to be solved to extract the solar cell parameters.

1 $$\frac{{d^{2} \psi }}{{dx^{2} }} = \frac{{\text{q}}}{\varepsilon }\left[ {p\left( x \right) - n\left( x \right) + N_{D}^{ + } \left( x \right) - N_{A}^{ - } \left( x \right) + p_{t} \left( x \right) - n_{t} \left( x \right)} \right]$$

2 $$- \frac{1}{q}\frac{{dJ_{n} }}{dx} + R_{n} \left( x \right) - G\left( x \right) = 0$$

3 $$\frac{1}{q}\frac{{dJ_{p} }}{dx} + R_{p} \left( x \right) - G\left( x \right) = 0$$

Here, ψ denotes the electrostatic potential, q is the electron charge, ε denotes the permittivity of the material, electron density and hole density are denoted as $p$ and $n$ respectively. ${N}_{A}^{-}$ and ${N}_{D}^{+}$ are the doping concentrations for acceptor and donor, ${p}_{t}$ is donor.

and ${n}_{t}$ is acceptor trap densities. ${J}_{n}$ and ${J}_{p}$ is electron and hole current density. G denotes the generation whereas ${R}_{n}$ and ${R}_{p}$ determines the net electron and hole recombination rate.

This simulation was carried out at a temperature of 300 K and illuminated light power of 1000 Wm^-2^ (AM1.5G spectrum) (Fig. 1d). This simulation tool can simulate a device with a maximum of seven different layers which limits the possibility of simulating a monolithic 2T tandem device. The detail procedure of simulating 2T tandem devices has been described in the supplementary. For 4T tandem devices, SCAPS was used to add the individual subcell $J-V$ and obtain 4T tandem $J-V$ characteristics.

## Results and discussion

### Optical characterization of the electrodeposited films

**Fig. 2:**
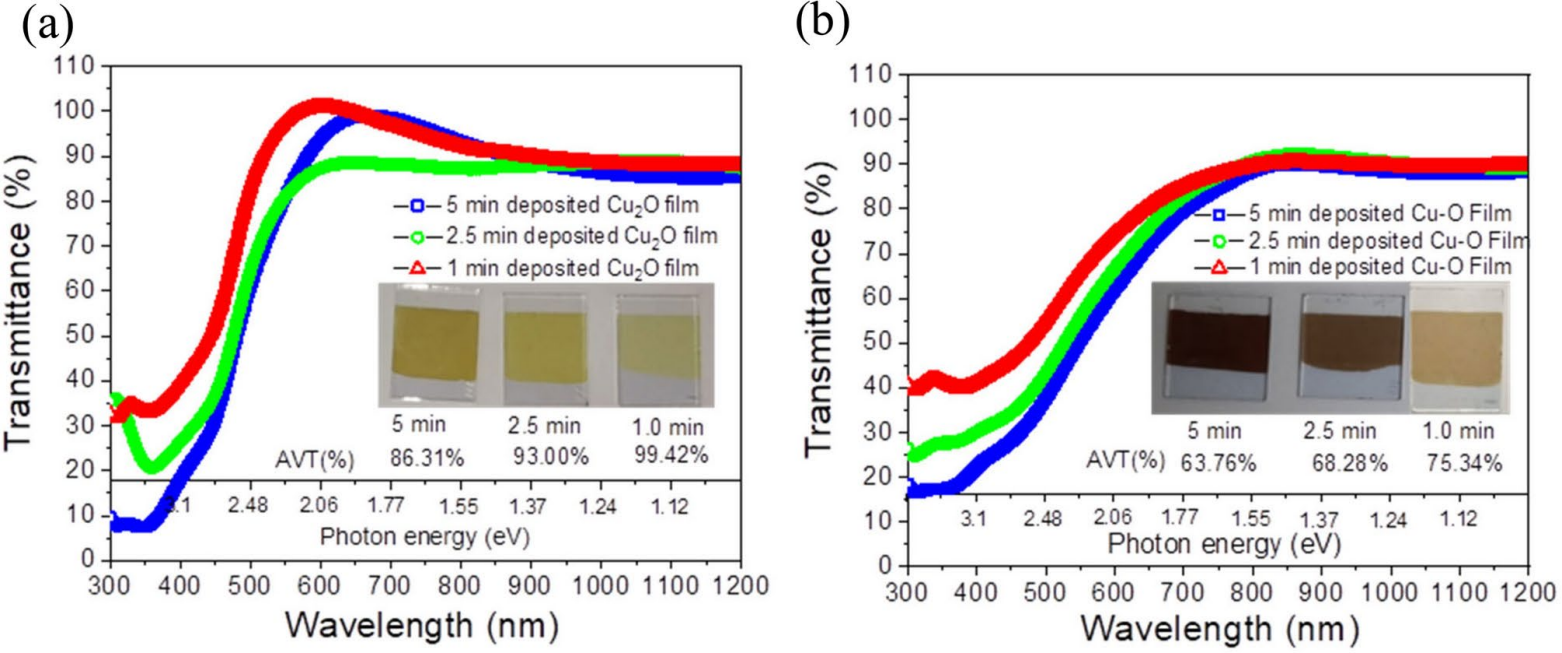
Transmittance spectra ($\%\text{T}$) as a function of wavelength $\left(\lambda \right)$ for (**a**) Cu_2_O films and (**b**) Cu–O films. Calculated AVT for different thickness films are shown in the inset along with the pictures of those films.

To access the suitability of the oxide films as HTL, transmittance (T) of the electrodeposited films of Cu_2_O and Cu–O films deposited for different period and having different thickness, were evaluated using UV–vis spectroscopic technique (Fig. 2a,b). The average visible light transmittance (AVT) was calculated according to the equation:

$$AVT = \frac{{\mathop \smallint \nolimits_{380\,{\text{nm}}}^{700\,{\text{nm}}} T\left( \lambda \right) \times P\left( \lambda \right) \times S\left( \lambda \right) d\lambda }}{{\mathop \smallint \nolimits_{380\,{\text{nm}}}^{700\,{\text{nm}}} P\left( \lambda \right) \times S\left( \lambda \right) d\lambda }}$$

where $T\left(\lambda \right)$ is the transmission spectrum, $P\left(\lambda \right)$ is the photopic response of the human eye and $S\left(\lambda \right)$ is the $AM1.5$ solar photon flux. AVT calculated for Cu_2_O films were in the range of 99.4% (for 1 min deposited film) to 86.3% (for 5 min deposited films) and it is much.

higher compared to Cu–O films. Above 700 nm wavelength, all the films are highly transparent ($T>90\%$) which corresponds to 1.77 eV bandgap energy. The optical constants, namely, refractive index (n) and extinction coefficient (k) was calculatd for Cu_2_O and Cu–O films (Supplementary Figure S3a). Extinction coefficient (k) was derived using the equation α = $\frac{4\pi k}{\lambda }$ . Here α is the absorption coefficient and it is given by the expression, $\alpha {\text{d = }} - {\text{ln}}\left( {\frac{{\text{T}}}{{{\text{1}} - {\text{R}}}}} \right)$ , where $d$ is the thickness, $T$ and $R$ are the transmittance and reflectance of the film respectively^43^. Refractive index (n) was calculated using Kramer-Kroenig relation^44^. These values of $n$ and $k$ were used as the input parameters for the transfer matrix method simulation to analyse the 2T tandem solar cell. The absorption coefficient ($\alpha$) was extracted from the UV–Vis absorbance data (Supplementary Figure S3b) for a known thickness of the film. Cross-sectional FESEM images confirmed thickness $\sim$ 160 nm for a 5-min deposited film (Supplementary Figure S3c). A linear fit at the band-edge revealed the bandgap energy ${(E}_{g})$ of 2.53 eV and 2.23 eV for Cu_2_O and Cu–O films respectively.

**Fig. 3:**
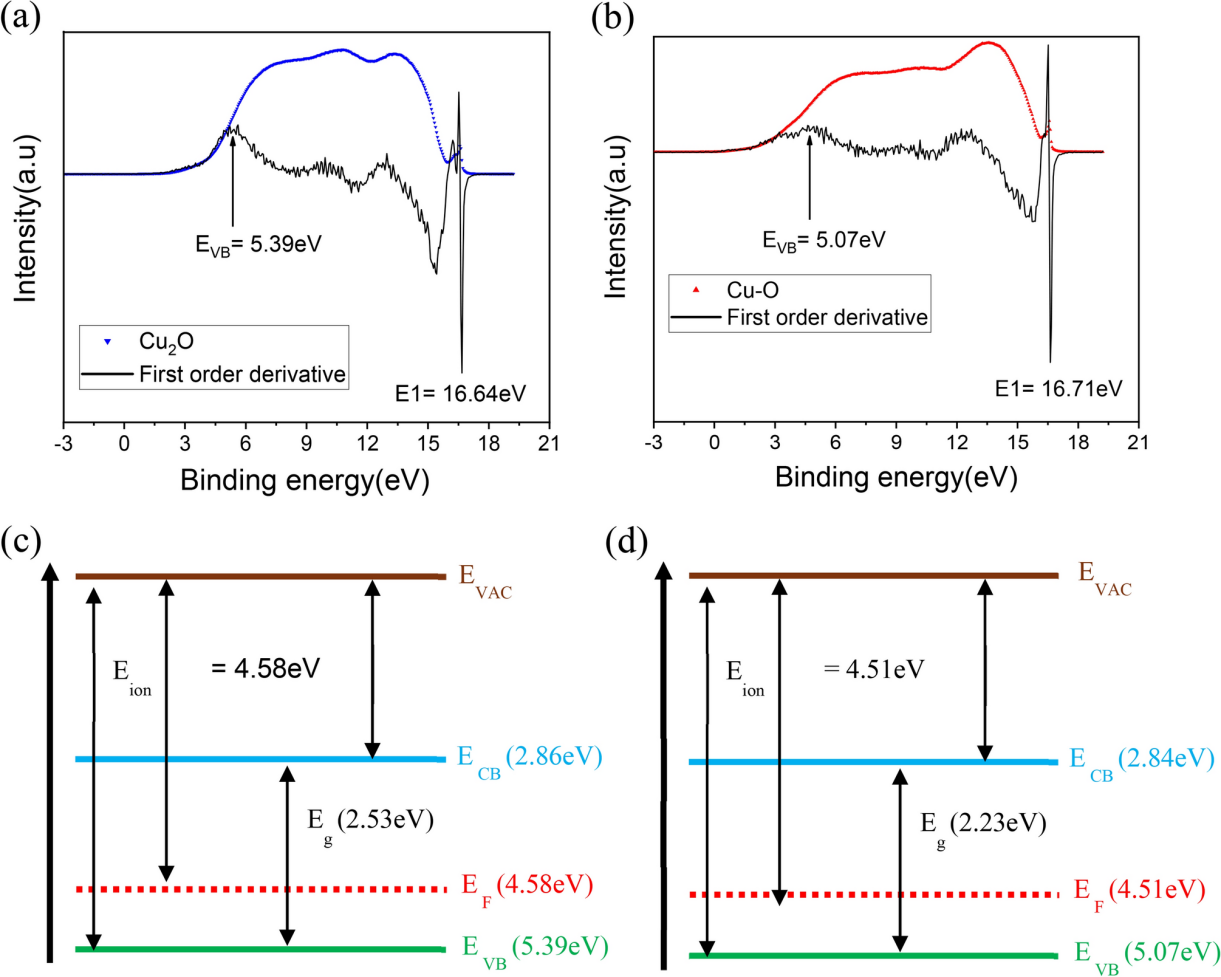
Ultraviolet photoelectron spectra for (**a**) Cu_2_O and (**b**) Cu–O along with the derivative spectra. The position of $E1$ and ${E}_{VB}$ has been identified using the inflection points in the derivative spectra. The calculated relative positions of the energy levels for (**c**) Cu_2_O and (**d**) Cu–O with respect to vacuum level.

Ultraviolet photoelectron spectroscopy (UPS) analysis was used to evaluate the valence band edge. The spectroscopic measurements were carried out using a Helium-I source with photon energy of 21.22 eV. Figures 3a and b show UPS spectra of Cu_2_O and Cu–O respectively. The onset energy of the secondary photoelectrons (E1) on the higher binding energy side was identified using a derivative of the UPS spectra which clearly reveals the “onset-points” at 16.64 eV and 16.71 eV for Cu_2_O and Cu–O, respectively. The work-function of these materials (Φ) or the position of the Fermi energy level, was calculated from the difference, ${E}_{F}=21.22-E1$. The valence band maximum (*E*_*VB*_) was identified clearly by the inflection point observed on the derivative spectra at the lower binding energy side. *E*_*VB*_ for Cu_2_O was at 5.39 eV and that for Cu–O was at 5.07 eV. Finally, the conduction band minima (*E*_*CB*_) position was calculated using the relation, ${E}_{CB}=({E}_{VB}-{E}_{g})$. All the calculated energy levels and their relative positions are depicted in Fig. 3c and d.

### Compositional analysis of the electrodeposited films

**Fig. 4:**
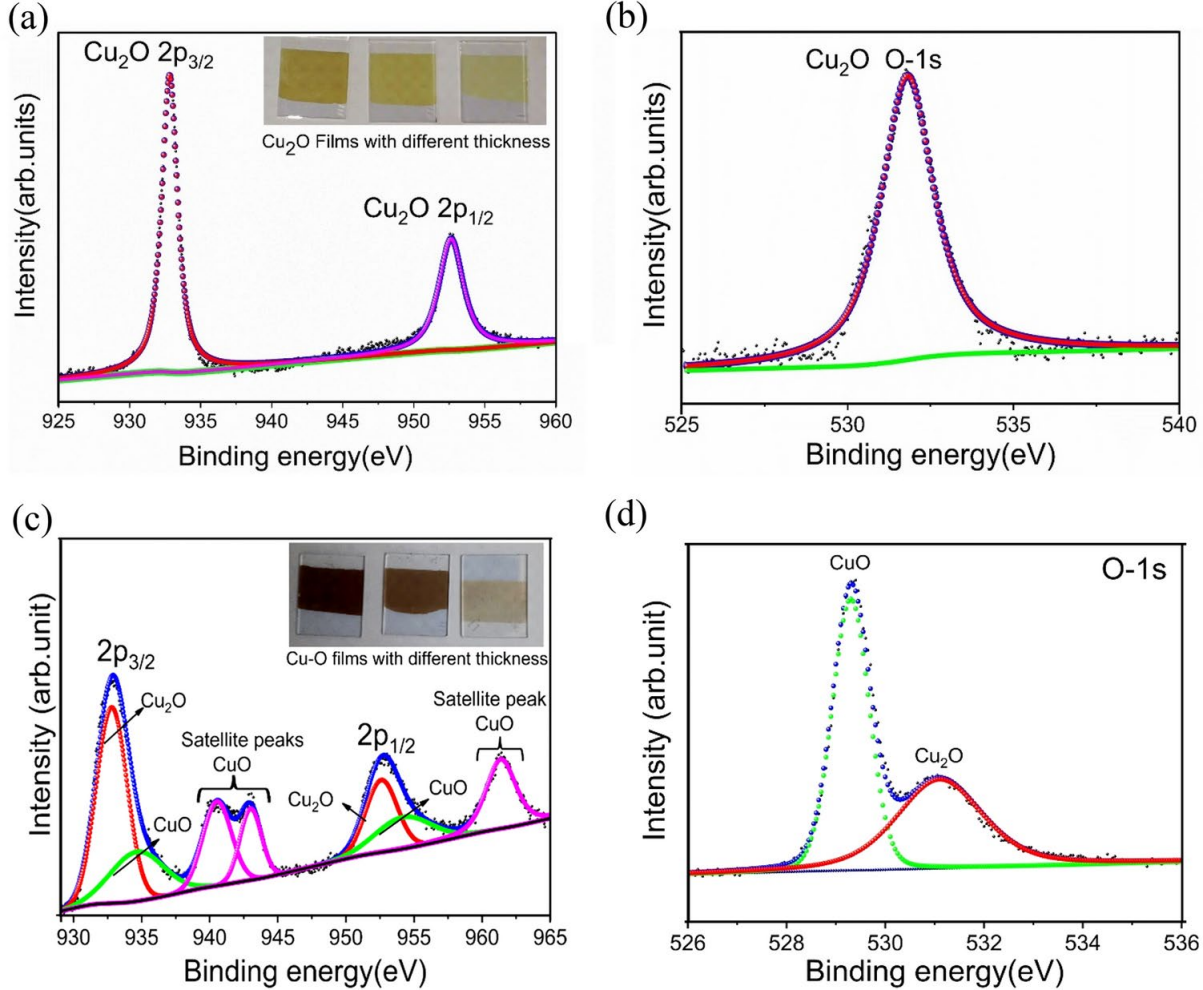
X-ray photoelectron spectroscopy (XPS) analysis showing (**a**, **c**) Cu 2*p* and (**b**, **d**) O 1*s* region for of electrodeposited Cu_2_O and air annealed Cu–O film.

The electrodeposited copper oxide films were analyzed by X-ray photoelectron spectroscopy (XPS) to investigate the surface elemental composition. The analysis of spectra was done using XPS PEAK FIT software, wherein Shirley’s method was used for the background corrections^45^. The deconvoluted high resolved spectra of Cu 2*p* and O-1* s* orbital of vacuum annealed (at 100 °C) Cu_2_O film and air annealed (at 300 °C) Cu–O film is shown in Fig. 4a–d. The Carbon 1* s* peak was found at 284.8 eV which is the standard value for the calibration purpose. The peak observed at 932.8 eV and 952.6 eV corresponds to Cu_2_O phase and peak at 934.6 eV and 954.2 eV corresponds to CuO phase which is assigned to 2*p*3/2 and 2*p*1/2 states of Cu^1+^ and Cu^2+^, respectively. The spin-orbital splitting of 19.8 eV is in accordance with the published literature confirming the Cu_2_O phase^43^. The high-resolution spectrum of Oxygen-1* s* indicates the existence of CuO phase at 529.30 eV and Cu_2_O phase at 531.1 eV. The satellite peaks or the shake up peaks at ~ 942 eV and ~ 961 eV are assigned to the Cu^2+^ state of CuO phase. Quantification of the relative ratio of Cu_2_O and CuO phase on the surface of the mixed phase Cu–O film was analyzed by evaluating the peak area ratio of Cu 2*p*3/2, Cu 2*p*1/2, and O 1*s.* The atomic percentage of the Cu_2_O and CuO states present in Cu–O film calculated without considering the shake-up peaks (summarized in Table S1) reveals a 1:1 ratio of both the species. The shake-up peak is absent in the case of pure Cu_2_O which could be due to filled orbital (d10) where there will be no photoelectron interaction with the valence band electron and no resultant columbic interaction.

### Simulation of ${\varvec{J}}-{\varvec{V}}$ characteristics of single junction cells with copper oxide HTL

#### Optimization of defect state density at HTL/perovskite interface

**Fig. 5:**
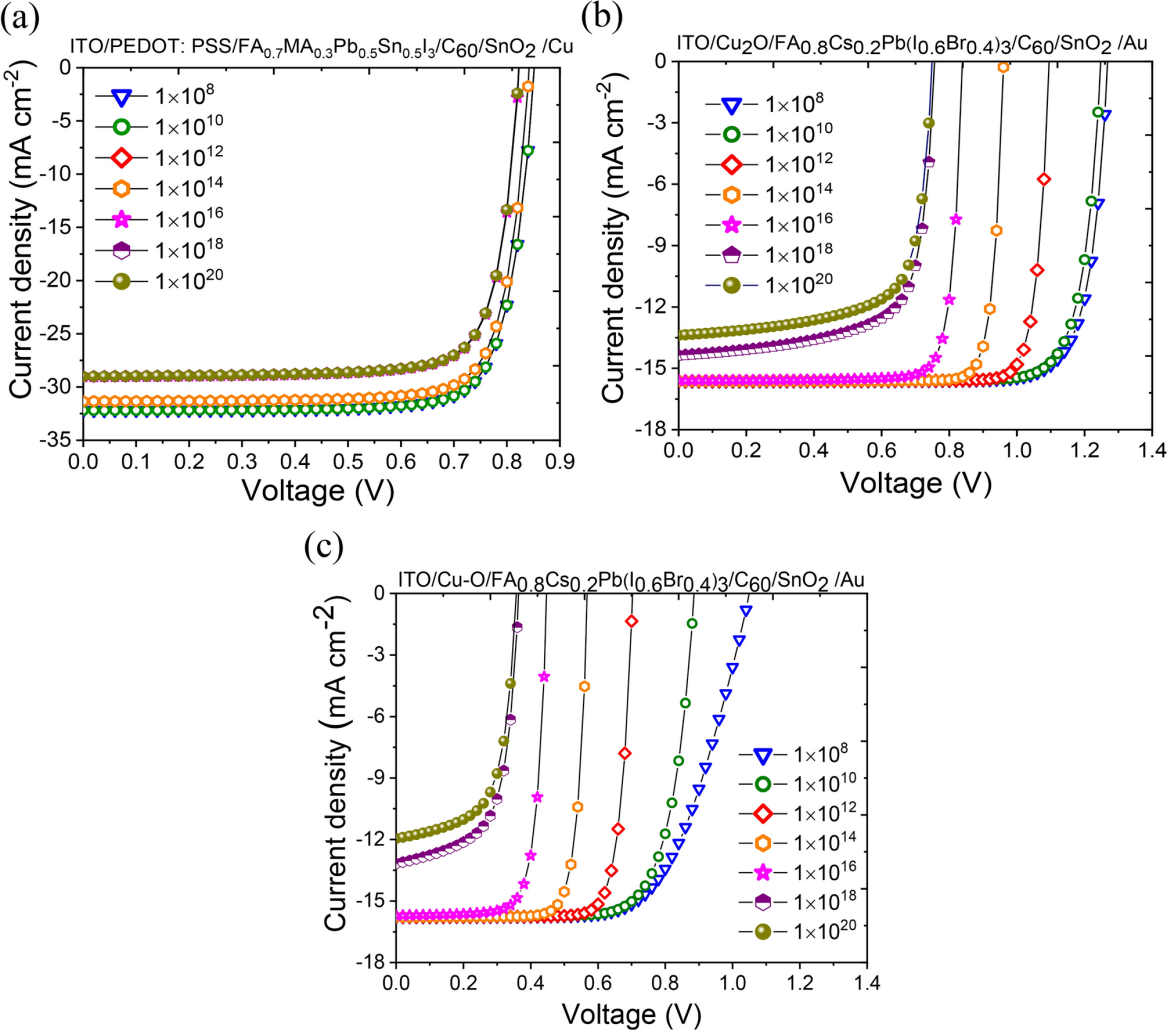
Current density versus voltage (J–V) characteristics obtained by varying interface defect between (**a**) PEDOT: PSS-HTL and a NBG perovskite (**b**) Cu_2_O-HTL and WBG perovskite and (**c**) Cu–O-HTL and WBG perovskite.

Single junction sub-cell configurations with WBG perovskite and NBG perovskite has been shown in Fig. 1a with three different HTL layer (viz. PEDOT:PSS, Cu–O and Cu_2_O). 1-D SCAPS simulation was performed on these device geometries to generate the corresponding $J-V$ characteristics. Here the light enters through the HTL/perovskite interface and most of the photons get absorbed at the vicinity of this interface. Hence the density of interface defects states plays a crucial role in the output characteristics of the device. To generate the best $J-V$ for the devices, interface defect density at the HTL/perovskite interface was varied from 1 × 10^8^ cm^−2^ to 1 × 10^20^ cm^−2^. The resulting curves are shown in Fig. 5a–c. Rest of the parameters required in this simulation were pre-optimized using a protocol described in Supplementary and tabulated in Supplementary Table S2. It is evident from the graphs that the best performance and a good match with experimental $J-V$ is achieved with interface defect density of ${10}^{10}$/cm^2^ for all the three single junction devices. Henceforth, for subsequent part of the simulations, the HTL/perovskite interface defect density was fixed at ${1\times 10}^{10}$/cm^2^.

#### Optimization of electron affinity of copper oxide HTL

**Fig. 6:**
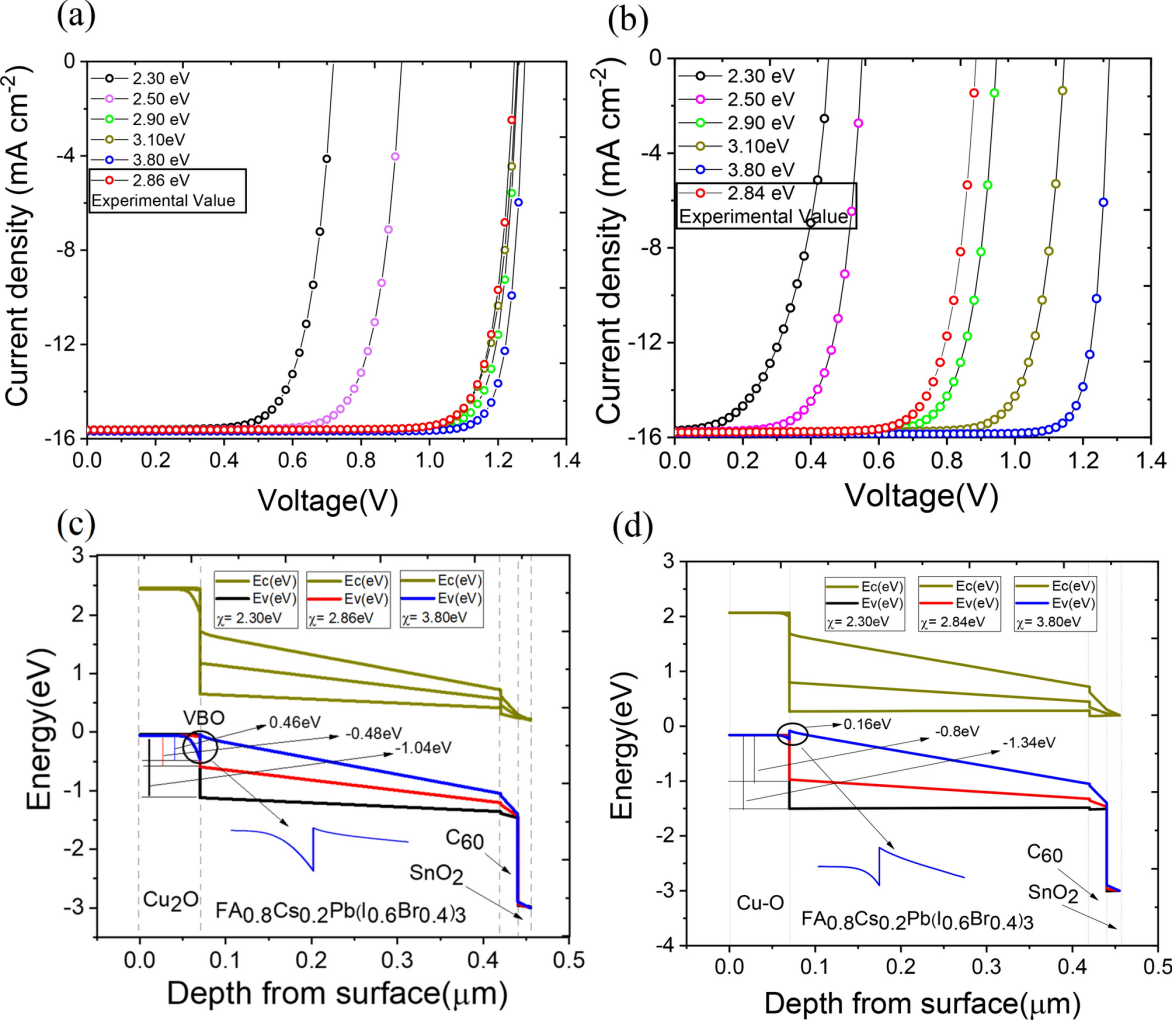
Current density versus voltage (J–V) characteristics for single junction solar cells with (**a**) Cu_2_O and (**b**) Cu–O HTL obtained by varying electron affinity (*χ*) of the HTLs. Energy band diagrams with valance band offset (VBO) at perovskite/HTL interface by varying electron affinity (*χ*) of (**c**) Cu_2_O and (**d**) Cu–O layers.

The proper optimization of energy band alignment of the perovskite and the charge transport layers can enhance the device performance through efficient extraction of the holes and electrons. Here, we have studied the effect of valence band offset (VBO) at the HTL/perovskite interface by varying the electron affinity of HTL materials, namely, Cu_2_O and Cu–O while the electron affinity of other layers was fixed at a particular value (Supplementary Table S2). Experimentally, the relative positions of band edges of the Cu_2_O film and the electron affinity can be modified by varying the pH of the electrolyte solution. Effect of variation of pH on the work function of the copper oxide film was systematically studied by Han, et al.^46^ (supplementary Table S4). In this work we varied the electron affinity $(\chi )$ of copper oxide layers in the range 2.30–3.80 eV. The experimentally obtained values of $\chi$ for Cu_2_O $\left(\approx 2.86 \,{\text{ eV}}\right)$ and Cu–O $\left(\approx 2.84 \,{\text{ eV}}\right)$ were also explored in the simulation. The results are depicted in Fig. 6a,b. With the increase in $\chi$, the VBO at the Cu_2_O/FA_0.8_Cs_0.2_Pb(I_0.6_Br_0.4_)_3_, interface decreased monotonically from $1.04 \,{\text{ eV}}$ to $0.48 \,{\text{ eV}}$. At $\chi =3.8 \,{\text{ eV}}$, VBO further reduces to $0.46 \,{\text{ eV}}$ with appearance of a kink (Fig. 6c). The solar cell parameters show marginal improvement when χ is varied from $\approx 2.86 \,{\text{ eV}}$ to $3.8 \,{\text{ eV}}$ for the devices, glass/ITO/Cu_2_O/FA_0.8_Cs_0.2_Pb(I_0.6_Br_0.4_)_3_ (${E}_{g}\approx 1.77 \,\text{eV},\text{WBG})$/C_60_/SnO_2_/Au. The parameters are listed in Table 1. The improvement can be attributed to the minor reduction in VBO and improved ohmic conduction at the interface.

**Table 1:** Summary of performance parameters of WBG single-junction sub-cells with two different HTL as calculated in the SCAPS simulation.

Device architecture	Electron affinity χ (eV)	V_OC_ (V)	J_SC_ (mA cm^−2^)	FF (%)	PCE (%)
ITO/Cu_2_O/FA_0.8_Cs_0.2_Pb(I_0.6_Br_0.4_)_3_ (${E}_{g}\sim 1.77\,\text{eV}$)(350 nm)/C_60_/SnO_2_ /Au	2.86	1.24	15.62	82.96	16.18
	3.8	1.27	15.69	86.54	17.36
ITO/Cu–O/FA_0.8_Cs_0.2_Pb(I_0.6_Br_0.4_)_3_ (${E}_{g}\sim 1.77\,\text{eV}$)(350 nm)/C_60_/SnO_2_/Au	2.84	0.88	15.78	75.72	10.59
	3.8	1.27	15.86	86.68	17.57

On the other hand, VBO at Cu–O/FA_0.8_Cs_0.2_Pb(I_0.6_Br_0.4_)_3_ interface decrease from $1.34 \,{\text{ eV}}$ to $0.8 \,{\text{ eV}}$ as $\chi$ increases from $2.30 \,{\text{ eV}}$ to the experimental value of $2.84 \,{\text{ eV}}$. When $\chi$ increases further to the value of $3.80 \,{\text{ eV}}$, the VBO drastically decreases to $0.16 \,{\text{ eV}}$ and a small kink appears at the interface (Fig. 6d). Consequently, the device with the configuration, glass/ITO/Cu–O/FA_0.8_Cs_0.2_Pb(I_0.6_Br_0.4_)_3_ (${E}_{g}\approx 1.77 \,\text{eV},\text{WBG})$/C_60_/SnO_2_ /Au shows an drastic improvement of efficiency from $10.59\%$ (when $\chi \sim 2.84 \,{\text{ eV}}$) to $17.56\%$ corresponding to $\chi \sim 3.8 \,{\text{ eV}}$ (Fig. 6b). In all the analysis so far, the thickness of the WBG perovskite layer was kept constant at 350 nm in accordance with the reported experimental results of Xiao et. al ^16^. In this simulation, the χ value corresponds to the electron affinity of a stand-alone copper oxide film in ultra-high vacuum condition. Under the actual working condition of the device (in an experiment) these energy levels can shift giving rise to Fermi level pinning effect. However, here our objective is to study the trend in the $J-V$ characteristics as a function of χ and draw a comparison between two types of oxides.

#### Effect of thickness of the perovskite absorber layer in the front and back cell

**Fig. 7:**
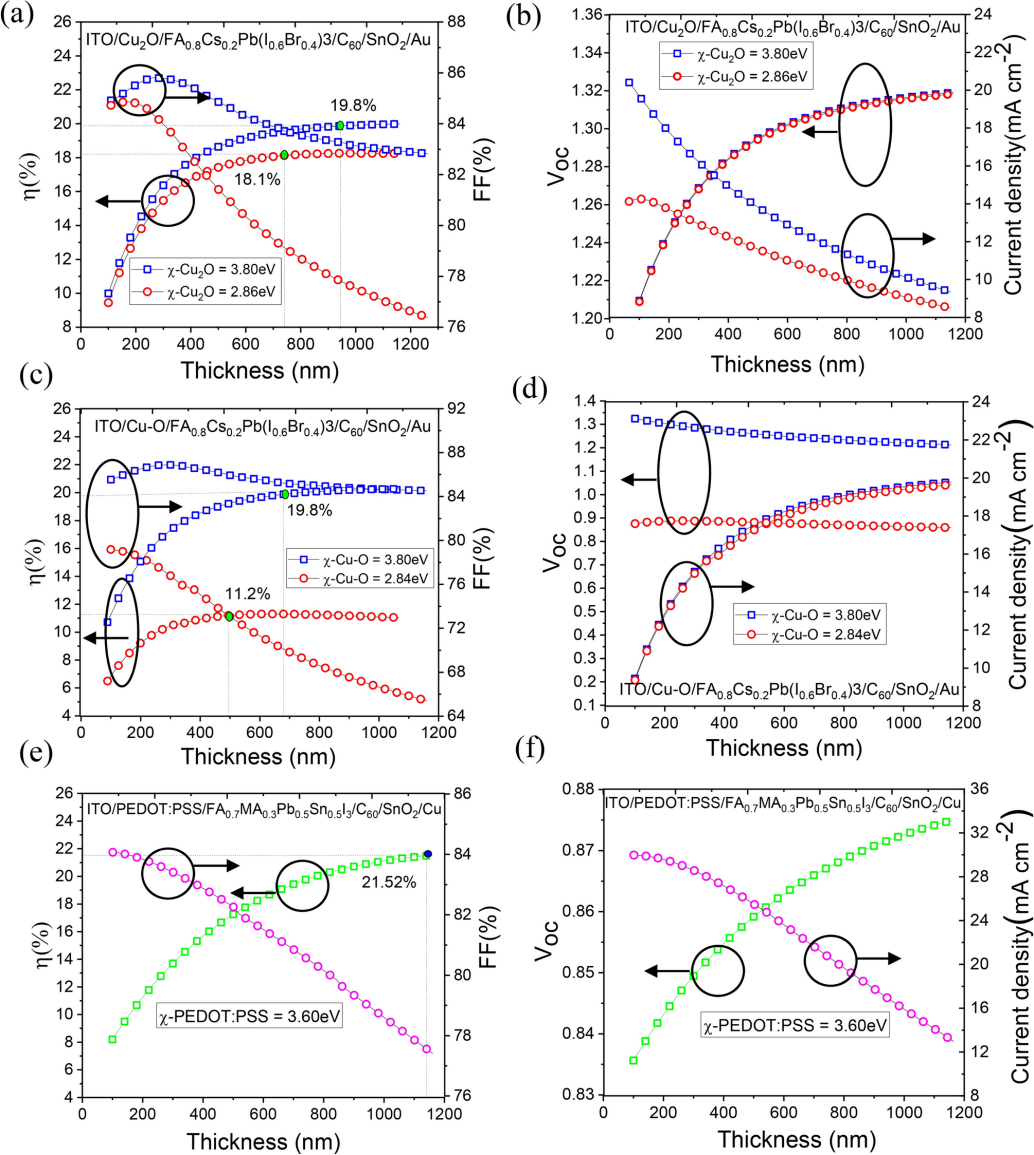
Thickness dependent solar cell parameters for (**a**), (**b**) Cu_2_O-based device with $\chi =2.86 \,{\text{ eV}}$ and 3.80eV, (**c**), (**d**) Cu–O-based device with $\chi = 2.84 \,{\text{ eV}}$ and 3.80eV and (**e**), (**f**) PEDOT: PSS-based device.

The power conversion efficiency of a tandem solar cell can be maximized by careful optimization of the thicknesses of the front and back sub-cells. Resultant short circuit current density $\left({J}_{SC}\right)$ of the tandem device is controlled mainly by the low-current producing sub-cell and hence it is often termed as “current-limiting” sub-cell. A close match between the ${J}_{SC}$ of the front sub-cell ( ${J}_{SC1}$) and back sub-cell ( ${J}_{SC2}$) is desired to ensure maximum efficiency of the tandem device. We explored the thickness dependence of solar cell parameters for both WBG single junction front sub-cell and NBG single junction back sub-cell and the results obtained in the simulation is shown in Fig. 7a–f. For the Cu_2_O-HTL/WBG-perovskite single junction cells (Fig. 7a), the PCE corresponding to $\chi =3.8 \,{\text{ eV}}$ is marginally higher than that for the experimental value of χ (2.86 eV). Although the ${J}_{SC}$ value is the same in both the cases, FF and ${V}_{OC}$ reduces as the thickness increases. The decrease in FF is more prominent for $\chi =2.86 \,{\text{ eV}}$. It can be attributed to higher VBO and consequent recombination of charge carriers at the HTL/perovskite interface. When the HTL material is Cu–O, the difference in the PCE for two different values of $\chi$ is quite drastic. The highest PCE value for WBG perovskite single junction subcell is $\approx 20\%$ corresponding to $\chi \approx 3.80 \,{\text{ eV}}$ and thickness of the perovskite layer $>500 {\text{nm}}$. However, as the electron affinity $\chi$ reduces to the experimental value the highest PCE reduces to only $10\%$. This large difference is mainly attributed to reduced $FF$ and ${V}_{OC}$. PCE values are more sensitive to the variation of $\chi$ for the Cu–O-HTL. For the NBG-perovskite back subcell with PEDOT:PSS- HTL, the simulation results yield a maximum PCE of 21.87% for the perovskite thickness of 1050 nm. These results match well with the experimental values as reported by Xiao et al.^16^

#### Construction of 2T tandem and predicting the maximum PCE with two different HTLs (Cu_2_O and Cu–O)

**Fig. 8:**
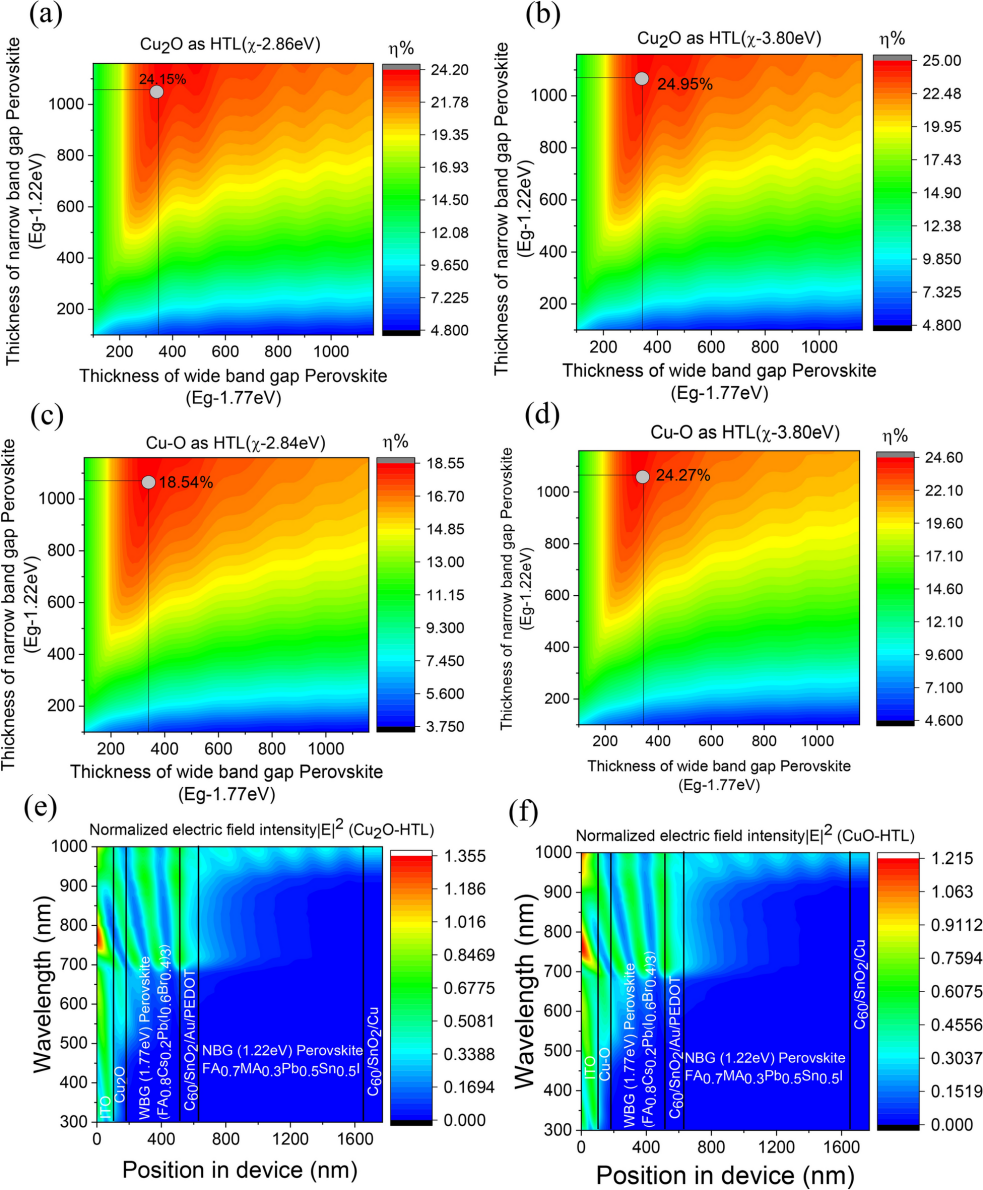
Power conversion efficiency *η *(in %) of 2T all perovskite device using (**a**). Cu_2_O with χ-2.86, (**b**) Cu_2_O with χ-3.80, (**c**) Cu–O with χ-2.84 and (**d**) Cu–O with χ-3.80 as HTL for front cell and PEDOT as HTL for back cell with varying thickness of perovskite layer. Electric field intensity distribution in (**e**) Cu_2_O and (**f**) Cu–O based tandem solar cell solar cell.

A tandem stack was constructed according to the configuration shown in Fig. 1b. Simulation was performed for both the electron affinity values, i.e. $$\chi =3.8 \,{\text{ eV}}$$ and the experimental values of $$\chi$$ for Cu_2_O and Cu–O. For best power conversion efficiency in a 2T tandem, the $${J}_{SC}$$ of the two sub-cells should have a close match ($${J}_{SC1}\approx {J}_{SC2})$$ which essentially means that the tandem works best for a particular combination of thicknesses of WBG and NBG perovskites with a complementary absorption spectra (Supplementary Figure S6) in the front and back cell, respectively. To find the optimum thickness combination, a detail optical simulation is performed. The methodology has been explained in detail in Supplementary section. In brief, transfer matrix method (TMM) simulation is used in combination with internal quantum efficiencies (IQE) to know the amount of current generated in each sub-cell inside a tandem stack as a function of front and back sub-cell thicknesses. These current values are then multiplied with the normalized form of the $$J-V$$ curves determined for a range of different layer thickness of both WBG and NBG perovskite layers. Finally, the $$J-V$$ curves of the front and back subcells are added to generate the $$J-V$$ curve of the whole 2T tandem stack. The predicted solar cell parameters ($${J}_{SC}, {V}_{OC}, FF)$$ for tandem cells as a function of front and back subcell thicknesses is shown in Supplementary section (Supplementary Figure S7a–d, Figure S8a–d and Figure S9a–d) for $$\chi =3.8 \,{\text{ eV}}$$ and the experimental values. The highest $$PCE$$ in the tandem, in all the cases is predicted for front cells with a thickness of 350 nm and back cells with thickness of 1050 nm (Fig. 8a–d). The performance parameters are summarized in Table 2. With the knowledge of optimised thickness of 350 nm and 1050 nm for front cell and back cell perovskite layer we have analysed electric field distribution in the Cu_2_O and Cu–O based tandem devices (Fig. 8e,f). The optical electric field is higher in front cell for shorter wavelengths whereas the back cell perovskite layer receives the longer part of the spectrum. The absorbed photon by the WBG front cell and NBG perovskite layer generate high density of charge carriers for both Cu_2_O and Cu–O based tandem cell (Supplementary Figure S10a and S10b).The charge carrier generation rate is described as Q = $${\alpha {I}_{0}e}^{-\alpha x}$$, where $${I}_{0}$$ is the intensity of the incident light and $$\alpha$$ is the absorption coefficient^47^. Because of the complimentary bandgap of the front and back cell perovskite the charge generation occurs in visible range (300–700 nm) and NIR ranges (700 to 900 nm),respectively.

**Table 2:** Summary of performance parameters of 2T tandem solar cell calculated using SCAPS and TMM simulation and comparison with experimentally reported data using NiO as HTL.

Device architecture	Type of analysis	HTL	Electron affinity χ (eV)	V_OC_ (V)	J_SC_ (mA cm^−2^)	FF (%)	PCE (%)
ITO/Cu_2_O/FA_0.8_Cs_0.2_Pb(I_0.6_Br_0.4_)_3_ (${E}_{g}\sim 1.77 \,{\text{ eV}}$) (350 nm)/C_60_/SnO2 /Au/PEDOT:PSS/FA_0.7_MA_0.3_Pb_0.5_Sn_0.5_I_3_ (${E}_{g}\sim 1.22 \,{\text{ eV}}$) (1050 nm)/C_60_/SnO_2_ /Cu	SCAPS + TMM	Cu_2_O	2.86	2.08	14.41	80.4	24.15
			3.8	2.11	14.44	81.7	24.95
ITO/Cu–O/FA_0.8_Cs_0.2_Pb(I_0.6_Br_0.4_)_3_ (${E}_{g}\sim 1.77 \,{\text{ eV}}$) (350 nm)/C_60_/SnO2 /Au/PEDOT:PSS/FA_0.7_MA_0.3_Pb_0.5_Sn_0.5_I_3_ (${E}_{g}\sim 1.22 \,{\text{ eV}}$) (1050 nm)/C_60_/SnO_2_ /Cu	SCAPS + TMM	Cu–O	2.84	1.72	13.96	78.00	18.54
			3.8	2.115	13.74	83.5	24.27
ITO/NiO/FA_0.8_Cs_0.2_Pb(I_0.6_Br_0.4_)_3_ (Eg-1.77 eV) (350 nm)/C_60_/SnO_2_/Au/PEDOT:PSS /FA_0.7_MA_0.3_Pb_0.5_Sn_0.5_I_3_(Eg-1.22 eV)(950–1150 nm)/C_60_/SnO_2_/Cu	Experimental	NiO		2.012	15.5	79.3	24.7 (24.2*)

*Certified efficiency.^16^

#### Construction of 4T tandem and predicting the maximum PCE with two different HTLs (Cu_2_O and Cu–O)

In a 4-teminal tandem device the two subcells with wide and narrow bandgap active layer are mechanically stacked on top of each other as shown in Fig. 1c. The front cell perovskite has a wide bandgap of 1.77 eV which is capable of absorbing shorter wavelength of light. While the remaining part of the spectrum (filtered light) is absorbed by the NBG-perovskite. The solar spectrum used for simulation is shown in Fig. 1d. Unlike 2T tandem, 4T tandem device does not require current matching between two subcells^48^. The simulation of the 4T device was carried using SCAPS-1D where single junction front cell, ITO/Cu_2_O or Cu–O/FA_0.8_Cs_0.2_Pb(I_0.6_Br_0.4_)_3_ (E_g_ ~ 1.77 eV)/C_60_/SnO_2_/ITO/Au was simulated for two different values of $\chi$ (i.e. 3.80 eV and the experimental values, 2.86 eV and 2.84 eV for Cu_2_O and Cu–O respectively) at a room temperature using AM 1.5G solar radiation The single junction back cell, ITO/PEDOT: PSS/FA_0.7_MA_0.3_Pb_0.5_Sn_0.5_I_3_ (E_g_ ~ 1.22 eV)/C_60_/SnO_2_/Cu was simulated with the filtered spectra S (λ) extracted following the equation: $S\left(\lambda \right)={S}_{0}\left(\lambda \right)\times \text{exp}({\sum }_{n=1}^{4}-({\alpha }_{n}(\lambda )\times {d}_{n}))$^49^ where ${S}_{0}\left(\lambda \right)$ is the standard AM 1.5G solar spectrum, d and α is the thickness and absorption coefficient (${\alpha }_{n}$) of the individual layers of the device. The PCE calculation of 4T tandem cell is explained schematically in supplementary section (Supplementary Figure S11) and a plot of PCE as a function of front and back subcell thickness has been shown in Supplementary Figure S12a–d. Since there is no recombination included in the simulation, the plot shows monotonically increasing PCE as the thicknesses of both sub cells increases. However, if we consider the optimized thickness combinations of 2T tandem, i.e., the combination of 350 nm and 1050 nm for front cell and back cell respectively, then a PCE value exceeding 32% seems achievable with copper oxide HTL. PCE is observed to be 34.98% and 35.51% in the devices with Cu_2_O-HTL analysed for χ = 2.86 and 3.80 eV respectively (Supplementary Figure S13a and S13b). Whereas Cu–O –HTL devices show a significant increase in the efficiency from 32.76 to 39.16% for χ − 2.84 eV and 3.80 eV, respectively (Supplementary Figure S13c and S13d). Device parameters for all the 4T devices are listed in Table 3.

**Table 3:** Summary of performance parameters of 4T tandem solar cell calculated using SCAPS and TMM simulation.

Device architecture		Electron affinity χ (eV)	V_OC_ (V)	J_SC_ (mA cm^−2^)	FF (%)	PCE (%)
ITO/Cu_2_O/FA_0.8_Cs_0.2_Pb(I_0.6_Br_0.4_)_3_ (Eg ~ 1.77 eV) (350 nm)/C_60_/SnO_2_ /ITO/Au	Front cell- AM1.5G spectra	2.86	1.25	15.65	84.26	16.58
		3.80	1.27	15.71	85.42	17.11
ITO/PEDOT: PSS/ FA_0.7_MA_0.3_Pb_0.5_Sn_0.5_I_3_ (Eg ~ 1.22 eV) /C_60_/SnO_2_/Cu	Back cell-filtered spectra	**–**	0.82	15.28	80.21	18.40
4T tandem device (Cu_2_O-HTL)	Mechanically stacked	2.86	**–**	**–**	**–**	34.98
		3.80	**–**	**–**	**–**	35.51
ITO/Cu–O/FA_0.8_Cs_0.2_Pb(I_0.6_Br_0.4_)_3_ (Eg ~ 1.77 eV) (350 nm)/C_60_/SnO_2_ /ITO/Au	Front cell- AM1.5G spectra	2.84	0.89	15.80	77.40	10.91
		3.80	1.27	15.88	85.55	17.31
ITO/PEDOT: PSS/ FA_0.7_MA_0.3_Pb_0.5_Sn_0.5_I_3_ (Eg ~ 1.22 eV) /C_60_/SnO_2_/Cu	Back cell-Filtered spectra	**–**	0.85	32.19	79.62	21.85
4T tandem device (Cu–O-HTL)	Mechanically stacked	2.84		**–**	**–**	32.76
		3.80	**–**	**–**	**–**	39.16

## Conclusion

In conclusion, we have demonstrated through detail optoelectronic simulation that both the phases of copper oxide namely Cu_2_O and Cu–O works efficiently as HTL in tandem solar cell and have the potential to replace PEDOT:PSS or the expensive materials like Spiro-OMeTAD. However, electron affinity and position of HOMO levels in these HTL-materials play an important role. Oxygen deficient phase such as, Cu_2_O is relatively difficult to achieve, most of the time annealing in uncontrolled ambient atmosphere results in a mixed phase copper oxide. Here we have demonstrated an easy and cost-effective method to achieve pure phase Cu_2_O and it also enabled us to compare between pure phase and mixed phase oxides of copper as a HTL in solar cell. The highest PCE predicted for 2T tandem devices (i.e. 24.95%) matches well with the experimental values (24.7%) indicating the reliability of the fitting parameters to replicate the realistic measurement conditions. Our simulation results show that, Cu_2_O works better compared to the Cu–O devices when the experimental values of χ is considered. However, if the value of χ is modified to 3.8 eV, the performance of Cu–O devices matches well with that of Cu_2_O devices. 4T tandem devices simulated with similar parameters indicates a monotonic increase in PCE as the thicknesses of front and back subcells increases. However, considering a similar thickness combination for front and back subcell as that of 2T tandem a PCE of 34.98% and 32.76% is predicted for Cu_2_O-HTL and Cu–O-HTL devices respectively. A highest PCE of 39.1% is predicted when χ increased to 3.80 eV for Cu–O-HTL device. Our study indicates that both Cu_2_O and Cu–O can potentially be used as environment friendly and cost-effective HTL layers for perovskite solar cells.

## Supplementary Information

Supplementary Information.

## Data Availability

The experimental and simulation data that support the findings of this study are deposited in the Materials Cloud archive (10.24435/materialscloud:kb-94).
