# Supplementary material for: Optoelectronic simulation and optimization of all perovskites tandem solar cells employing electrodeposited copper oxide as hole transport layer
Source: Sci Rep. 2025 Mar 22;15:9916. doi: 10.1038/s41598-025-93982-7 (PMC11929914; doi:10.1038/s41598-025-93982-7)
Supplement: Supplementary file 1 — Supplementary Information. [file 41598_2025_93982_MOESM1_ESM.docx]

**Optoelectronic simulation and optimization of all perovskites tandem solar cell employing electrodeposited copper oxide as hole transport layer**

Vishwas D. Patel, Dhritiman Gupta*

Department of Physics, School of Advanced Science (SAS), Vellore Institute of

Technology (VIT), Vellore-632014, Tamil Nadu, India.

Corresponding Author

*Email: dhritiman.gupta@vit.ac.in; Tel: +919047509685

(b)

(a)


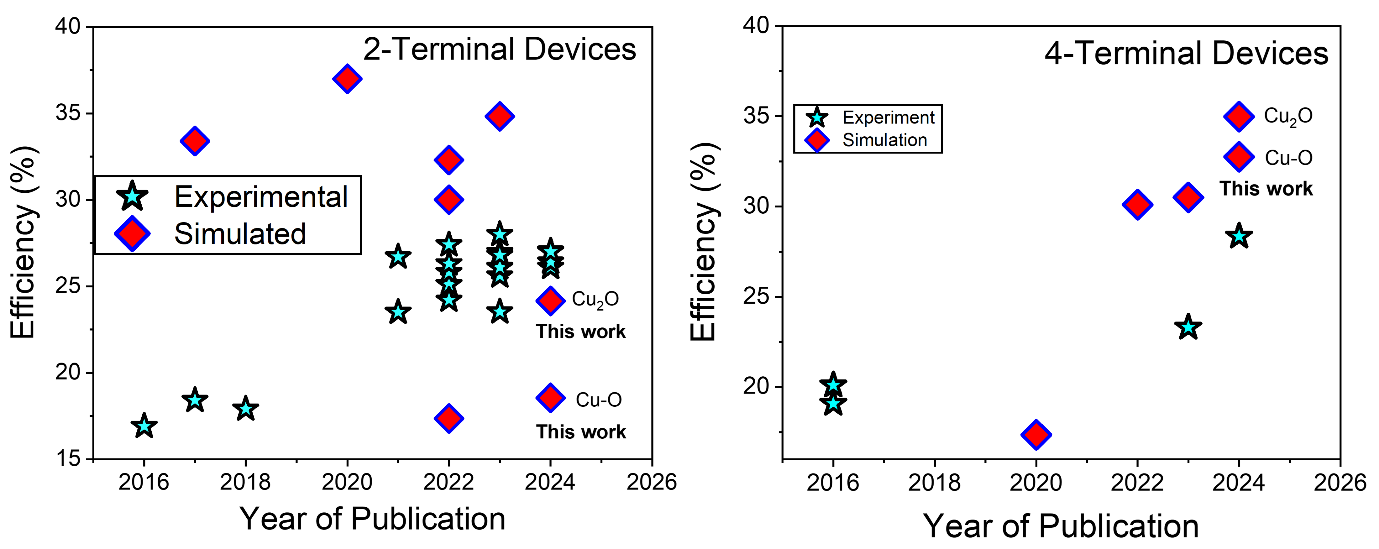


***Figure S1:*** *Summery graph representing efficiencies of fabricated and simulated perovskite/perovskite (a)2-T and (b) 4-T tandem solar using metal oxide as HTL^[[1]](#endnote-1)-,^ ^[[2]](#endnote-2),^ ^[[3]](#endnote-3),^ ^[[4]](#endnote-4),^ ^[[5]](#endnote-5) ,^^[[6]](#endnote-6),^ ^[[7]](#endnote-7),^ ^[[8]](#endnote-8),^ ^[[9]](#endnote-9) ,^ ^[[10]](#endnote-10) ,^**^[[11]](#endnote-11),^ ^[[12]](#endnote-12),^ ^[[13]](#endnote-13),^* *^[[14]](#endnote-14) ,^^[[15]](#endnote-15),^* *^[[16]](#endnote-16),^ ^[[17]](#endnote-17),^ ^[[18]](#endnote-18),^ ^[[19]](#endnote-19),^ ^[[20]](#endnote-20),^ ^[[21]](#endnote-21),^ ^[[22]](#endnote-22),^ ^[[23]](#endnote-23),^ ^[[24]](#endnote-24),^^[[25]](#endnote-25),^ ^[[26]](#endnote-26),^^[[27]](#endnote-27),^ ^[[28]](#endnote-28),^ layer in last few years and displaying the same for our simulated devices using experimental χ of 2.86 for Cu_2_O and 2.84eV Cu-O .*


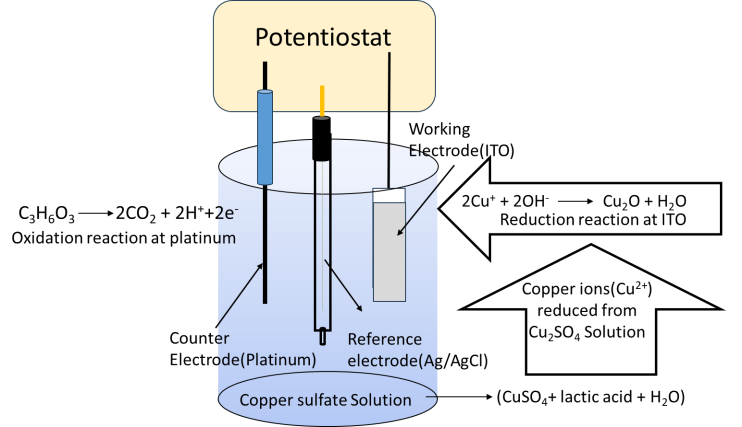


(b)

(a)


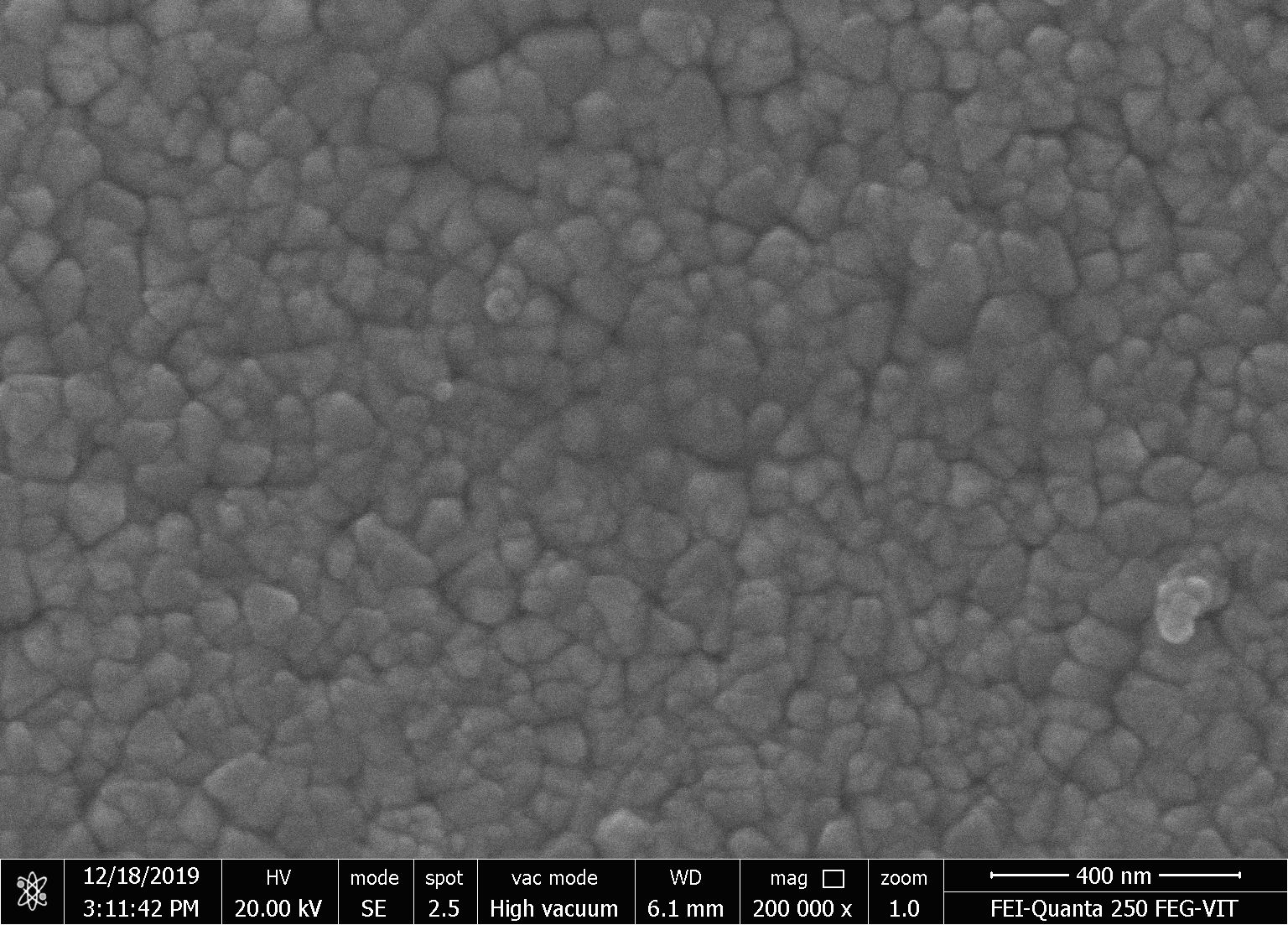

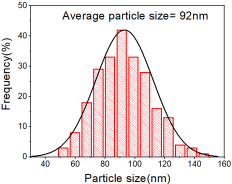

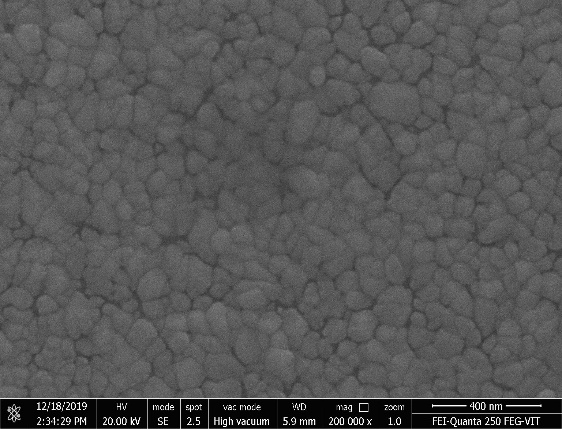

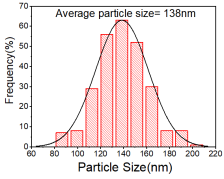


(c)

***Figure S2:*** *(a) Electrodeposition setup (CHI-601C) with three different electrodes used for deposition of Cu_2_O thin film for different thicknesses. Schematic diagram of the Electrodeposition set up and FESEM image revealing the surface morphology of (b) Cu_2_O and (c) Cu-O layer shows a uniform and compact layer with an average particle size of ~92 nm and ~138 nm.*

(b)

(a)

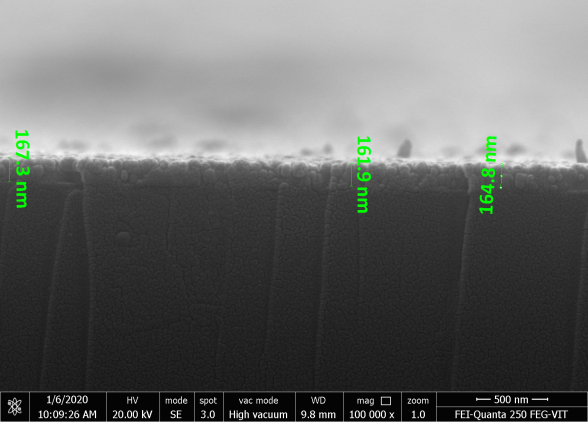


(c)

***Figure S3***: *(a) The optical constants(a)refractive index(n) and extinction coefficient(k) for Cu_2_O and Cu-O film. (b) Tauc plot [(αh**υ)^2^ (eVcm^-1^)^2^ Vs Energy (eV), α (cm^-1^) is the absorption coefficient] to extract the optical bandgap of the copper oxides. To locate the band-edge, the first derivative was plotted (inset) and the inflection points at the lower energy side has been marked to signify the band-edge positions. (c) The cross-sectional FESEM image of Cu_2_O film shows a thickness of ~160 nm.*

***Table S1: -*** Summary of the relative fractions of Cu_2_O and Cu-O phases present on the surface of the film, extracted from XPS data

| **Cu_2_O**  **Peaks** | Percentage (%) | **CuO**  **Peaks** | Percentage (%) |
| --- | --- | --- | --- |
| **Cu2p3/2** | 48.74±32.2 | **Cu2p3/2** | 51.25±33.3 |
| **Cu2p1/2** | 43.46±42.4 | **Cu2p1/2** | 56.53±49.2 |
| **O1s** | 53.84±4 | **O1s** | 46.15±1.7 |

**Methodology for Simulation of 2T Tandem in SCAPS-1D:**

**1)** **Optimization of simulation parameters and generating single junction** $\boldsymbol{J-V}$ **curves**: We simulated in SCAPS the single junction solar cells with the same device configuration as reported by Xiao et. al. (glass/ITO/NiO_x_/VNPB/FA_0.8_Cs_0.2_Pb(I_0.6_Br_0.4_)_3_ /C_60_ /SnO_2_ /Au and ITO/PEDOTPS/FA_0.7_MA_0.3_Pb_0.5_Sn_0.5_I C_60_/SnO_2_/Cu) where NiO_x_ and PEDOT was used as HTL and obtained the best fit with the reported experimental data (Figure S4a,b). Parameters optimized in this way are listed in Table S2 [referred as “Optimized”] (ii) In the next step, Cu-O and Cu_2_O was introduced as bottom HTL replacing NiO_X_, (iii) Single junction $J-V$ characteristics of front and back cells were generated via SCAPS-simulation for varying thicknesses of perovskite layers. Parameters used in this step are shown in Table S2. Some of the parameters were optimized from previous steps and some were obtained from literature. We plugged in our own experimental values wherever appropriate. $J-V$ characteristics obtained this way has been discussed in the main manuscript.

(a)

(b)

***Figure S4:*** *Experimental* $J-V$ *characteristics of single junction (a) wide band gap front-cell and (b) narrow band gap back cell with NiO_X_* and PEDOT as *HTL, respectively as reported by Xiao et. al. (Open symbols) and their corresponding simulated* $J-V$ *characteristics (solid line) in SCAPS-1D. Via optimizing the parameters a best fit was achieved for front cell thickness of 500 nm and back cell thickness of 1050 nm*

***Table S2:*** *Comparison between experimental and simulated single junction solar-cell performance parameters for WBG and NBG perovskite sub-cells with NiO*_x_ *and PEDOT:PSS HTL layers respectively. The experimental values were reported by Xiao et. al.^29^*

| **Device Architecture** | **Type of Analysis** | **V_OC_ (V)** | **J_SC_**  **(mA cm^-2^)** | **FF (%)** | **PCE (%)** |
| --- | --- | --- | --- | --- | --- |
| ITO/PEDOT:PSS/FA_0.7_MA_0.3_Pb_0.5_Sn_0.5_I_3_  (Eg -1.22eV)/C_60_/SnO_2_/Cu | Experiment | 0.85 | 31.6 | 80.80 | 21.7 |
| ITO/PEDOT:PSS/FA_0.7_MA_0.3_Pb_0.5_Sn_0.5_I_3_  (Eg -1.22eV)/C_60_/SnO_2_/Cu | 1D-SCAPS | 0.85 | 32.19 | 79.62 | 21.85 |
| ITO/NiO/FA_0.8_Cs_0.2_Pb(I_0.6_Br_0.4_)_3_ (Eg-1.77eV)) (500nm)/C_60_/ SnO_2_/Au | Experiment | 1.20 | 17.10 | 77.9 | 15.90 |
| ITO/NiO/FA_0.8_Cs_0.2_Pb(I_0.6_Br_0.4_)_3_ (Eg-1.77eV)) (500nm)/C_60_/ SnO_2_/Au | 1D-SCAPS | 1.15 | 16.94 | 77.52 | 15.18 |

| **Parameters** | **Layers** | | | | | | | |
| --- | --- | --- | --- | --- | --- | --- | --- | --- |
|  | **Cu_2_O** | **Cu-O** | **NiO** | **PEDOT: PSS** | **Perovskite**  **E_g_-1.77eV** | **Perovskite**  **E_g_-1.22eV** | **C_60_** | **SnO_2_** |
| Thickness  (nm) | 70  [*] | 70  [*] | 20  [^[[29]](#endnote-29)^] | 30  [Optimized] | Varying | Varying | 20 | 15 |
| Energy band gap  (E_g_ in eV) | 2.53  [*] | 2.23  [*] | 2.8  [^[[30]](#endnote-30)^] | 1.50  [Optimized] | 1.77  [29] | 1.22  [29] | 1.70  [^[[31]](#endnote-31)^] | 3.20  [33] |
| Electron affinity  (χ in eV) | 2.86  [*] | 2.84  [*] | 2.50  [^[[32]](#endnote-32)^] | 3.60  [^[[33]](#endnote-33)^] | 4.10  [Optimized] | 4.10  [Optimized] | 4.20  [33] | 4.20  [33] |
| Relative dielectric permittivity (ε_r_) | 7.60  [^[[34]](#endnote-34)^, ^[[35]](#endnote-35)^] | 10.260  [^[[36]](#endnote-36)^] | 11.90  [^[[37]](#endnote-37)^] | 3.00  [33] | 24.10[^[[38]](#endnote-38)^] | 24.10  [Optimized] | 4.00  [33] | 9.00  [^[[39]](#endnote-39)^] |
| Effective Density of states at valence band (N_V_ in cm^-3^) | 1.0×10^19^  [34] | 5.50×10^19^  [^[[40]](#endnote-40)^] | 5.50×10^20^  [Optimized] | 1.0×10^18^  [Optimized] | 1.0×10^19^  [Optimized] | 1.0×10^18^  [Optimized] | 1.80×10^19^  [39] | 1.80×10^19^  [33,39] |
| Effective Density of states at conduction band (N_C_ in cm^-3^) | 2.43×10^19^  [34] | 2.20×10^19^  [40] | 2.20×10^19^  [Optimized] | 1.00×10^17^  [Optimized] | 1.0×10^19^  [Optimized] | 1.0×10^18^  [Optimized] | 2.20×10^18^  [12] | 2.20×10^18^  [33,39] |
| Hole mobility  (μ_p_ in cm^2^.V^−1^s^−1^) | 100  [34] | 100  [40] | 50  [Optimized] | 100  [33] | 55  [Optimized] | 60  [Optimized] | 30  [33] | 10  [33] |
| Electron mobility  (μ_n_ in cm^2^.V^−1^s^−1^) | 50  [34] | 0.1  [40] | 1.00×10^-5^ | 4  [33] | 55  [Optimized] | 60  [Optimized] | 50  [33] | 20  [33] |
| Uniform shallow donor doping  (N_D_ in cm^-3^) | - | - | - | - | 8.00×10^18^  [Optimized] | 1.00×10^18^  [Optimized] | 1.00×10^16^  [39] | 1.00×10^15^  [39] |
| Uniform shallow acceptor doping  (N_A_ in cm^-3^) | 1.00×10^18^  [Optimized] | 1.00×10^18^  [Optimized] | 1.00×10^18^  [Optimized] | 1.00×10^18^  [33] | 8.00×10^18^  [Optimized] | 1.00×10^18^  [Optimized] | - | - |
| Defect density  of the material  (N_t_ in cm^-3^) | 5.00×10^16^  [Optimized] | 1.00×10^18^  [Optimized] | 1.00×10^18^  [Optimized] | 1.00×10^15^  [33] | 1.00×10^15^  [Optimized] | 2.00×10^15^  [Optimized] | 1.00×10^15^  [39] | 1.00×10^15^  [39] |

***Table S3****: - Simulated parameter used in SCAPS-1D for perovskite/perovskite tandem solar cell*

†Hole thermal velocity and Electron thermal velocity of all the layer was kept at fix value of 1.00×10^7^ cm/s,

*Experimental values obtained through our own measurements on electrodeposited copper oxide (as discussed in main text)

**Table S4: -** Summary of changes in work function of copper oxide as a function of change in pH value of the electrolyte solution as reported by Han et. al. [Ref. No. 46 in main text] and comparison with our work.

| **pH** | **Band Gap (eV)** | **Work function (eV)**  **UPS-analysis** | **This work**  **(pH of electrolyte solution was kept constant at 11)** | | |
| --- | --- | --- | --- | --- | --- |
|  |  |  | **Film** | **Band Gap (eV)** | Work function (eV) |
| 7 | 1.82 | 4.32 |  |  |  |
| 9 | 1.86 | 4.97 |  |  |  |
| 11 | 1.93 | 5.15 | Cu_2_O | 2.53 | 4.58 |
| 12 | 1.90 | 5.23 |  |  |  |

**2) Optimization of front cell and back cell thickness for 2T tandem device:**

There is a limitation in number of layers in a stack tant can be used in SCAPS-1D simulation (which is around seven). Hence a full $J-V$ simulation under AM1.5 illumination of a monolithic 2T tandem solar cell with 11 layers is not possible in SCAPS. We adopted a method that is well documented and previously reported by us. In the beginning the internal quantum efficiencies (IQE) of the WBG and NBG single junction perovskite solar cells are calculated. This is done by dividing the $J_{SC}$ obtained from SCAPS-1D simulation with the $\left( J_{SC} \right)_{max}$ obtained from transfer matrix simulation. This exercise is done for both the sub- cells with varied thickness of the perovskite layers. Then after the IQE as a function of thickness of the perovskite layer for both NBG and WBG subcells with different HTL layers.

Next, the complete 2T tandem stack with 11 layers is simulated using transfer matrix simulation to find the maximum photon absorption in individual perovskite layer. Assuming zero recombination loss, the absorbed photon density can be treated as maximum $J_{SC}$ generated in front and back subcells. This $J_{SC}$ multiplied by the $IQE$ of individual subcells will give rise to the actual $J_{SC}$ generated in the front and back subcell of the tandem. We then use the $J-V$ characteristics of individual single junction subcells and normalize their $J_{SC}$. These normalized $J-V$ characteristics are then multiplied with the $J_{SC}$ of the subcells inside the tandem. Thus, we obtain the $J-V$ characteristics of the each subcells inside the tandem. Finally, these the $J-V$ characteristics are added following the principle that we add the voltage for each current. The whole process flow has been shown in the schematic (***Figure S5***).

Schematic presentation of 2-T device simulation process process

&

Maximum J_sc-TMM_ & Maximum J_sc-SCAPS_

Single junction front cell (varying thickness of WBG PVK) & back cell (varying thickness of NBG PVK)

Optical Constants (n &k)

and

Solar AM1.5G spectra

Input data to TMM

IQE (*d1, d2*) =$\frac{Jsc-SCAPS}{Jsc-TMM}$

$J_{SC-Tandem}(d1)=IQE(d1)\times J_{SC}(Front cell TMM-Full stack)$

$$J_{SC-Tandem}(d2)=IQE(d2)\times J_{SC}(Back cell TMM-Full stack)$$

Normalized *J-V* obtained from SCAPS-1D for both cells (thickness series)

Input parameters for the python script

2-T solar cell parameters

for the optimized thickness of front cell and back cell.

***Figure S5:*** *Flow chart describing the 2-T device simulation process*

***Figure S6****: Absorption profile of perovskite layer for the optimized thickness of 350nm and 1050nm for WBG Perovskite (1.77eV) and NBG Perovskite(1.22eV), calculated using TMM.*

(b)

(a)

******

(d)

(c)

***Figure S7****: -Current density(J_sc_)of 2-T all perovskite device using(a)Cu_2_O with χ-2.86 (b)Cu_2_O with χ-3.80 (c) Cu-O with χ-2.84 and (d)Cu-O with χ-3.80 as HTL for front cell wide and PEDOT as HTL for back cell with varying thickness of wide bang gap and narrowband gap perovskite layer.*

(b)

(a)

(d)

(c)

***Figure S8****: -Open circuit voltage (V_oc_)of 2-T all perovskite device using(a)Cu_2_O with χ-2.86 (b)Cu_2_O with χ-3.80 (c) Cu-O with χ-2.84 and (d)Cu-O with χ-3.80 as HTL for front cell wide and PEDOT as HTL for back cell with varying thickness of wide bang gap and narrowband gap perovskite layer.*

(a)

(b)

(c)

(d)

***Figure S9****: -Fill Factor(%)of 2-T all perovskite device using(a)Cu_2_O with χ-2.86 (b)Cu_2_O with χ-3.80 (c) Cu-O with χ-2.84 and (d)Cu-O with χ-3.80 as HTL for front cell wide and*

*PEDOT as HTL for back cell with varying thickness of wide bang gap and narrowband gap perovskite layer.*

(b)

(a)

**

***Figure S10****: TMM simulated charge generation rate in (a) Cu_2_O and (b) Cu-O based perovskite/perovskite tandem solar cell*

Filtered Spectra

4-T Tandem


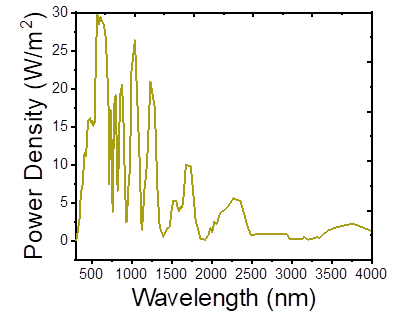


AM 1.5G Spectra

**Front cell**- ITO/Cu_2_O or Cu-O (χ -2.86 & 3.80eV) /FA_0.8_ Cs_0.2_Pb (I_0.6_Br_0.4_)_3_(Eg-1.77eV) (Varying thickness (**T1**) -100 to 1100nm)/C_60_/SnO_2_ /ITO/Au


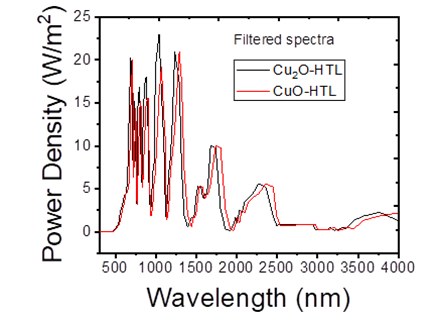


**Back cell**- ITO/PEDOT: PSS/FA_0._MA_0.3_Pb_0.5_ Sn_0.5_ I_3_ (Eg -1.22eV) (Varying thickness (**T2**) -100 to 1100nm)/C_60_/SnO_2_/Cu

**Simulation of both subcell with SCAPS-1D**

**Front cell** PCE (η) - F1(100nm)....F21(1100nm) **Back cell** PCE (η) – B1(100nm)…..B21(1100nm)

*step size 50nm

**Tandem 4-T-η (**Figure S12)

$$\left[ \begin{matrix} F1+B1 & \cdots& F21+B1 \\ \vdots& \ddots& \vdots\\ F1+B21 & \cdots& F21+B22 \end{matrix} \right]$$

Schematic presentation of 4-T device simulation process process

***Figure S11:*** *Flow chart describing the 4-T device simulation process*

(b)

(a)

(d)

(c)

***Figure S12****: -Power conversion efficiency (η(%)) of 4-T all perovskite device using(a)*

*Cu_2_O with χ-2.86 (b)Cu_2_O with χ-3.80 (c) Cu-O with χ-2.84 and (d)Cu-O with χ-3.80 as HTL for front cell wide and PEDOT as HTL for back cell with varying thickness of wide bang gap and narrowband gap perovskite layer.*

(a)

(b)

(c)

(d)

***Figure S13:*** *Current density versus voltage (J-V) characteristics of 4-T tandem solar cell device with (a) Cu_2_O χ = 2.86eV (b) Cu_2_O χ = 3.80eV and (c) Cu-O χ = 2.84eV (d) Cu-O χ = 3.80eV*

***Figure S14:-*** *Current density versus voltage (J-V) characteristics of experimentally fabricated single junction perovskite device with the device configuration ITO/Cu-O/Cs_0.05_(FA_x_MA_1-x_)_0.95_PbI_y_Br_3-y_/PCBM/BCP/Au*

**References**

1. . Eperon, G. E. et al. Perovskite-perovskite tandem photovoltaics with optimized band gaps. *Science* *354*(6314), 861-865(2017). https://doi.org 10.1126/science.aaf971 (2016). [↑](#endnote-ref-1)
2. . Rajagopal, A. et al. Highly efficient perovskite–perovskite tandem solar cells reaching 80% of the theoretical limit in photovoltage. *Advanced materials*, *29*(34), 1702140(2017). https://doi.org/10.1002/adma.201702140 [↑](#endnote-ref-2)
3. . Li, C. et al. Thermionic Emission–Based Interconnecting Layer Featuring Solvent Resistance for Monolithic Tandem Solar Cells with Solution‐Processed Perovskites. Advanced Energy Materials 8(36), 1801954(2018). https://doi.org/10.1002/aenm.201801954 [↑](#endnote-ref-3)
4. . Gao, H. et al. Thermally stable all‐perovskite tandem solar cells fully using metal oxide charge transport layers and tunnel junction. *Solar RRL* *5*(12), 2100814(2021). https://doi.org/10.1002/solr.202100814 [↑](#endnote-ref-4)
5. . Xiao, K. et al. Scalable processing for realizing 21.7%-efficient all-perovskite tandem solar modules. *Science*, *376*(6594),762-767(2022). https://doi.org/10.1126/science.abn769 [↑](#endnote-ref-5)
6. . Wu, P. et al. Efficient and Thermally Stable All‐Perovskite Tandem Solar Cells Using All‐FA Narrow‐Bandgap Perovskite and Metal‐oxide‐based Tunnel Junction. *Advanced Energy Materials* 12(48), 2202948(2022).https://doi.org/10.1002/aenm.202202948 [↑](#endnote-ref-6)
7. . Chen, H. et al. Regulating surface potential maximizes voltage in all-perovskite tandems. *Nature* *613* (7945), 676-681(2023). https://doi.org/10.1038/s41586-022-05541-z [↑](#endnote-ref-7)
8. . Li, T., Xu, J., Lin, R. et al. Inorganic wide-bandgap perovskite subcells with dipole bridge for all-perovskite tandems. *Nat. Energy* 8, 610–620 (2023). https://doi.org/10.1038/s41560-023-01250-7 [↑](#endnote-ref-8)
9. . Yu, D., Pan, M., Liu, G. et al. Electron-withdrawing organic ligand for high-efficiency all-perovskite tandem solar cells. *Nat Energy* 9, 298–307 (2024). https://doi.org/10.1038/s41560-023-01441-2 [↑](#endnote-ref-9)
10. . Jiang, X., et al.Surface heterojunction based on n-type low dimensional perovskite film for highly efficient perovskite tandem solar cells. *National Science Review*, nwae055(2024). https://doi.org/10.1093/nsr/nwae055 [↑](#endnote-ref-10)
11. .Singh, A., & Gagliardi, A. Efficiency of all-perovskite two-terminal tandem solar cells: A drift-diffusion study. *Solar Energy* 187, 39-46 (2019) https://doi.org/10.1016/j.solener.2019.05.006. [↑](#endnote-ref-11)
12. .Soldera, M., Koffman-Frischknecht, A., & Taretto, K. Optical and electrical optimization of all-perovskite pin type junction tandem solar cells. *Journal of Physics D: Applied Physics* 53(31), 315104(2020). https://doi.org/10.1088/1361-6463/ab8851 [↑](#endnote-ref-12)
13. . Yadav, S., et al. Optoelectronic modeling of all-perovskite tandem solar cells with design rules to achieve> 30% efficiency. *Solar Energy Materials and Solar Cells* *242*, 111780 (2022). https://doi.org/10.1016/j.solmat.2022.111780 [↑](#endnote-ref-13)
14. . Madan, J., Pandey, R., & Sharma, R. Device simulation of 17.3% efficient lead-free all-perovskite tandem solar cell. *Solar energy* 197, 212-221(2020). https://doi.org/10.1016/j.solener.2020.01.006 [↑](#endnote-ref-14)
15. . Hörantner, M. T., et al. The potential of multijunction perovskite solar cells. *ACS Energy Letters* 2(10), 2506-2513(2017). https://doi.org/10.1021/acsenergylett.7b00647 [↑](#endnote-ref-15)
16. . Shankar, G., Kumar, P., & Pradhan, B. All-perovskite two-terminal tandem solar cell with 32.3% efficiency by numerical simulation. *Materials Today Sustainability* *20*, 100241(2022). https://doi.org/10.1016/j.mtsust.2022.100241 [↑](#endnote-ref-16)
17. . Wang, W., Yu, G., & Attique, S. Dragon Mimic Shape Facilitate Ultrahigh‐Performance Flexible All‐Perovskite Tandem Solar Cells. *Solar RRL* *7*(7), 2201064(2023). https://doi.org/10.1002/solr.202201064 [↑](#endnote-ref-17)
18. . Li, H., Wang, Y., Gao, H. et al. Revealing the output power potential of bifacial monolithic all-perovskite tandem solar cells. *Elight* 2, 21(2022). https://doi.org/10.1186/s43593-022-00028-w [↑](#endnote-ref-18)
19. . Qiao, L. et al. Crystallization Enhancement and Ionic Defect Passivation in Wide‐Bandgap Perovskite for Efficient and Stable All‐Perovskite Tandem Solar Cells. *Advanced Functional Materials* *34*(7), 2308908(2024). https://doi.org/10.1002/adfm.202308908 [↑](#endnote-ref-19)
20. . Lee, S., et al. Buried interface modulation via PEDOT: PSS ionic exchange for the Sn-Pb mixed perovskite based solar cells. *Chemical Engineering Journal*, *479*, 147587 (2024). [↑](#endnote-ref-20)
21. . Li, S., et al. A Generic Strategy to Stabilize Wide Bandgap Perovskites for Efficient Tandem Solar Cells. *Advanced Materials* *36*(9), 2307701(2024). https://doi.org/10.1016/j.cej.2023.147587 [↑](#endnote-ref-21)
22. . Yu, D., Pan, M., Liu, G. et al. Electron-withdrawing organic ligand for high-efficiency all-perovskite tandem solar cells. Nat Energy 9, 298–307 (2024). https://doi.org/10.1038/s41560-023-01441-2 [↑](#endnote-ref-22)
23. . Gao, H. et al. Homogeneous crystallization and buried interface passivation for perovskite tandem solar modules. *Science* *383*(6685), 855-859(2024). https://doi.org/10.1126/science.adj608 [↑](#endnote-ref-23)
24. . Xie, Z., Chen, S., Pei, Y., Li, L., Zhang, S., & Wu, P. Enhanced efficiency in two-terminal all-perovskite tandem solar cells via binary functional high polymer doping strategy. *Chemical Engineering Journal* *482*, 148638(2024). https://doi.org/10.1016/j.cej.2024.148638 [↑](#endnote-ref-24)
25. . He, R., Wang, W., Yi, Z. et al. Improving interface quality for 1-cm2 all-perovskite tandem solar cells. Nature 618, 80–86 (2023). https://doi.org/10.1038/s41586-023-05992-y [↑](#endnote-ref-25)
26. . Moradbeigi, M., Razaghi, M. Investigation of optical and electrical properties of novel 4T all perovskite tandem solar cell. *Sci Rep* 12, 6733 (2022). https://doi.org/10.1038/s41598-022-10513-4 [↑](#endnote-ref-26)
27. . Luo, X. et al. Theoretical Analysis of All‐Inorganic Wide Bandgap Perovskite/Sn‐Based Narrow Bandgap Perovskite Tandem Solar Cells. *Solar RRL* *7*(10),2300081(2023). https://doi.org/10.1002/solr.202300081 [↑](#endnote-ref-27)
28. . Jia, P. et al. Intermediate Phase Suppression with Long Chain Diammonium Alkane for High Performance Wide‐Bandgap and Tandem Perovskite Solar Cells. *Advanced Materials*, 2400105(2024). https://doi.org/10.1002/adma.202400105 [↑](#endnote-ref-28)
29. . Xiao, K., Lin, R., Han, Q. et al. All-perovskite tandem solar cells with 24.2% certified efficiency and area over 1 cm2 using surface-anchoring zwitterionic antioxidant. Nat Energy 5, 870–880 (2020). https://doi.org/10.1038/s41560-020-00705-5 [↑](#endnote-ref-29)
30. . Singh, A. and Gagliardi, A. Efficiency of all-perovskite two-terminal tandem solar cells: A drift-diffusion study. *Solar Energy* *187*, 39-46(2019). https://doi.org/10.1016/j.solener.2019.05.006 [↑](#endnote-ref-30)
31. Golubev, T., Liu, D., Lunt, R. and Duxbury, P. Understanding the impact of C_60_ at the interface of perovskite solar cells via drift-diffusion modeling. *AIP Advances* *9*(3), 035026(2019). https://doi.org/10.1063/1.5068690 [↑](#endnote-ref-31)
32. . Tekin, S.B. et al. Electron affinity of metal oxide thin films of TiO_2_, ZnO, and NiO and their applicability in 28.3 THz rectenna devices. *Journal of Applied Physics* *134*(8), 084503 (2023). https://doi.org/10.1063/5.0157726 [↑](#endnote-ref-32)
33. . Kumar, D. et al. Numerical Study on the Effect of Dual Electron Transport Layer in Improving the Performance of Perovskite–Perovskite Tandem Solar Cells. *Advanced Theory and Simulations* *6*(3),2200800(2023). https://doi.org/10.1002/adts.202200800 [↑](#endnote-ref-33)
34. . Sekkat, A. et al. Unveiling Key Limitations of ZnO/Cu_2_O All-Oxide Solar Cells through Numerical Simulations. *ACS Applied Energy Materials* *5*(5), 5423-5433(2022). https://doi.org/10.1021/acsaem.1c03939 [↑](#endnote-ref-34)
35. . Yang, Y., Xu, D., Wu, Q. *et al.* Cu_2_O/CuO Bilayered Composite as a High-Efficiency Photocathode for Photoelectrochemical Hydrogen Evolution Reaction. *Sci Rep* 6, 35158 (2016). https://doi.org/10.1038/srep35158 [↑](#endnote-ref-35)
36. . Nakaoka, K., Ueyama, J. and Ogura, K. Photoelectrochemical Behavior of Electrodeposited CuO and Cu_2_O Thin Films on Conducting Substrates. *Journal of the Electrochemical Society* *151*(10), C661(2004). https://doi.org/10.1149/1.1789155 [↑](#endnote-ref-36)
37. . Rao, K.V. and Smakula, A.Dielectric Properties of Cobalt Oxide, Nickel Oxide, and Their Mixed Crystals. *Journal of Applied Physics* *36*(6), 2031-2038(1965). https://doi.org/10.1063/1.1714397 [↑](#endnote-ref-37)
38. . Brivio, F., Butler, K.T., Walsh, A. and Van Schilfgaarde, M. Relativistic quasiparticle self-consistent electronic structure of hybrid halide perovskite photovoltaic absorbers. *Physical Review B* *89*(15),155204(2014). https://doi.org/10.1103/PhysRevB.89.155204 [↑](#endnote-ref-38)
39. . Shankar, G., Kumar, P. and Pradhan, B. All-perovskite two-terminal tandem solar cell with 32.3% efficiency by numerical simulation. *Materials Today Sustainability* *20*, 100241(2022). https://doi.org/10.1016/j.mtsust.2022.100241 [↑](#endnote-ref-39)
40. . Sawicka-Chudy, P. et al.Simulation of TiO_2_/CuO solar cells with SCAPS-1D software. *Materials Research Express* *6*(8),085918(2019).https://doi.org/10.1088/2053-1591/ab22aa [↑](#endnote-ref-40)
